# European Society of Clinical Pharmacy definition of the term clinical pharmacy and its relationship to pharmaceutical care: a position paper

**DOI:** 10.1007/s11096-022-01422-7

**Published:** 2022-06-06

**Authors:** Tobias Dreischulte, Bart van den Bemt, Stephane Steurbaut, the European Society of Clinical Pharmacy

**Affiliations:** 1 Institute of General Medicine, University Hospital of Ludwig-Maximilians-University Munich, Pettenkoferstrasse. 8a, 80336 Munich, Germany; 2 Department of Pharmacy, Sint Maartenskliniek, Nijmegen, The Netherlands; 3 Department of Pharmacy, Radboud University Medical Centre, Nijmegen, The Netherlands; 4 Centre for Pharmaceutical Research, Research Group of Clinical Pharmacology and Clinical Pharmacy, Vrije Universiteit Brussel, Laarbeeklaan 103, 1090 Jette, Belgium; 5 Department of Hospital Pharmacy, UZ Brussel, Laarbeeklaan 101, 1090 Jette, Belgium

**Keywords:** Clinical Pharmacy, Definition, European Society of Clinical Pharmacy, Pharmaceutical care

## Abstract

Many definitions of the term clinical pharmacy exist, but a number of ambiguities remain. In order to clarify the European Society of Clinical Pharmacy (ESCP) position on what defines clinical pharmacy, a consultation exercise was conducted among ESCP members with the findings used as the basis for an updated definition. The updated definition clarifies that clinical pharmacy (1) represents both a professional practice and field of research, (2) aims to optimise the utilisation of medicines in order to achieve person-centered and public health goals, (3) as a practice encompasses cognitive, managerial and interpersonal activities targeting all stages of the medicines use process, and as a field of research generates knowledge that informs clinical decision-making, health care organisation or policy, (4) as a practice is restricted to pharmacists, (5) can be practiced regardless of setting, and (6) encompasses pharmaceutical care but is not restricted to it.

**Supplementary Information:**

The online version contains supplementary material available at 10.1007/s11096-022-01422-7.

## Introduction: The need for an updated definition of clinical pharmacy

Since the 1950s, pharmacists across the globe have recognized the need to shift the focus of pharmacy practice from the development, manufacture and supply of medicines towards ensuring the appropriate use of medicines to improve patient outcomes. The terms ‘clinical pharmacy’ and ‘pharmaceutical care’ have been instrumental in initiating a shift towards a more person-centered approach. However both terms are open to interpretation and as a consequence neither has been fully understood and used uniformly [1]. Clear definitions of both terms are prerequisites to their uniform interpretation and efficient communication within and outside the pharmacy profession [2].

In 2013, the Pharmaceutical Care Network Europe (PCNE) published an updated definition of pharmaceutical care in response to ongoing uncertainty around the term [3]. In the case of clinical pharmacy, a series of definitions have emerged since the early 1960s when the term first appeared in the literature [4–12]. However, the need for an updated definition of the term is supported by a survey of pharmacists affiliated with the European Society of Clinical Pharmacy (ESCP) conducted in 2014, which revealed uncertainties around the scope of clinical pharmacy activities, providers, settings and its relationship to pharmaceutical care [13].

In order to clarify these and other questions around the term clinical pharmacy, ESCP has looked to update and optimise its own definition through an iterative process. In this commentary, we describe this work and present the updated ESCP definition with supporting rationale.

## Consultation exercise to update the ESCP definition of clinical pharmacy

**Fig. 1:**
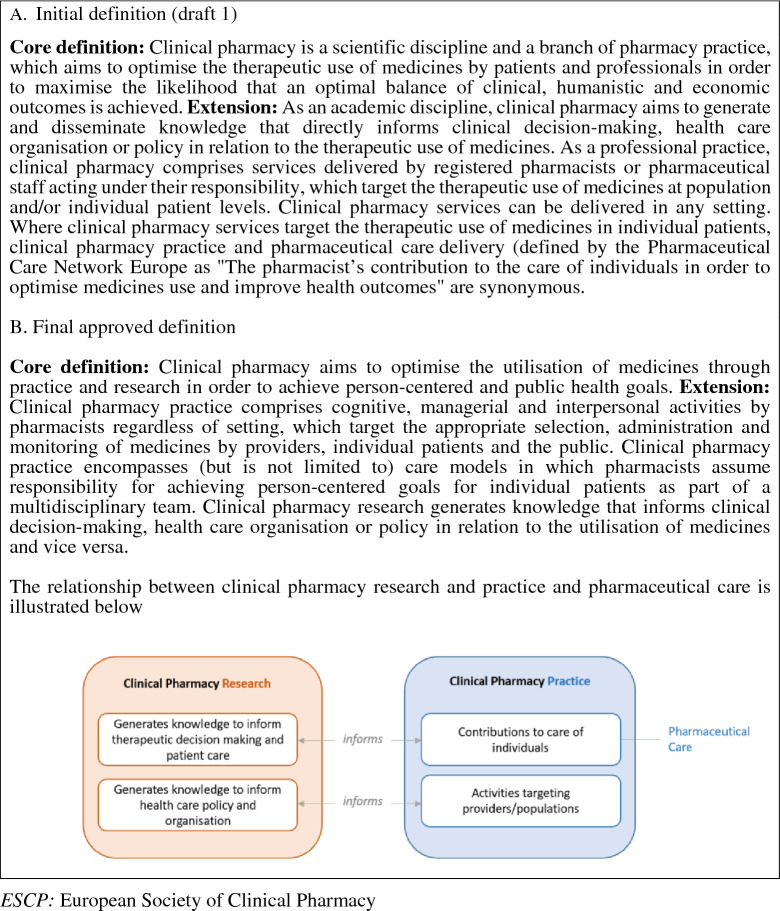
Initial and final iteration of the ESCP definition. *ESCP* European Society of Clinical Pharmacy

Informed by an ESCP survey in 2014, we drafted a definition of clinical pharmacy with accompanying rationale, and emailed this in April 2018 to all registered ESCP members at the time (*n* = 263) to get a formative view from participants [13]. The first draft of the definition is shown in Fig. 1 and the survey and accompanying rationale are available as supplementary electronic material S1. The survey asked participants to rate (yes, uncertain, no) (1) whether the draft definition provided clarity on six key questions, and (2) to which extent participants agreed with the view expressed within the draft definition after reading the accompanying rationale. In the case of an ‘uncertain’ or ‘no’ answer, participants were asked for an explanation for their answer in free text. A total of 89 (response rate 33.8%) ESCP members from across 25 different European countries completed the online survey. The main findings are presented in Table 1, with 65/89 (73%) participants also providing a total of 185 free text comments (available as supplementary electronic material S2).

**Table 1:** Findings of the consultation exercise

Questions	Responses			
Considering the proposed definition (including the extension) without the rationale: Does the definition provide a clear answer to the question …	Total count	Yes count (%)	Uncertain count (%)	No count (%)
As to whether the term clinical pharmacy refers to a professional practice and/or a scientific discipline?	89	73 (82.0)	11 (12.4)	5 (5.6)
As to which professional activities clinical pharmacy encompasses?	89	69 (77.5)	14 (15.7)	6 (6.7)
As to who can provide clinical pharmacy services?	89	62 (69.7)	22 (24.7)	5 (5.6)
In which settings clinical pharmacy services can be provided?	89	69 (77.5)	13 (14.6)	7 (7.9)
Which outcomes clinical pharmacy aims to achieve?	89	74 (83.1)	9 (10.1)	6 (6.7)
Whether and how the term clinical pharmacy differs from the term pharmaceutical care?	88	52 (59.1)	21 (23.9)	15 (17.0)
After reading the rationale: Do you agree with the view expressed on …				
Whether clinical pharmacy refers to a professional practice, a scientific discipline, or both?	86	76 (88.4)	7 (8.1)	3 (3.5)
Which professional activities clinical pharmacy encompasses?	85	71 (83.5)	9 (10.6)	5 (5.9)
Who can provide clinical pharmacy services?	86	68 (79.1)	10 (11.6)	8 (9.3)
The question in which settings clinical pharmacy services can be provided?	86	64 (74.4)	13 (15.1)	9 (10.5)
Which outcomes clinical pharmacy aims to achieve?	86	75 (87.2)	8 (9.3)	3 (3.5)
Whether and how clinical pharmacy differs from pharmaceutical care?	86	61 (70.9)	18 (20.9)	7 (8.1)

Between 18 and 41% of participants remained uncertain regarding the six questions posed after reading the draft definition (without considering the rationale). After reading the rationale, the level of disagreement with the draft definition was generally low, with the highest being in relation to the setting of clinical pharmacy provision (10.5%), possible clinical pharmacy providers (9.3%), and the clinical pharmacy-pharmaceutical care relationship (8.1%).

## The updated definition

Based on the findings of the consultation exercise, a further draft of the definition of clinical pharmacy and rationale was produced by one of the authors (TD). The two other authors (BvB and SS) commented independently on whether the free text comments made by participants on the initial draft definition were resolved, not resolved or partially resolved in a revised definition (Fig. 1) with further amendments made based on consensus between all three authors (supplementary electronic material S2). In the second version, 133 (72%) comments were judged to be resolved, 26 (14%) partially resolved and 26 (14%) not resolved. Further comments on the second version were invited and received from the ESCP GC-RESC (joint General Committee-Research, Education, Special Interest Group and Communication Committee). After a final round of minor amendments, the final revised definition and rationale were approved by the ESCP GC on 20/04/2020.

## Rationale for the revised definition

The following paragraphs provide the rationale for the approved revised definition (Fig. 1) with reference to six key questions.

**1. Is clinical pharmacy a scientific discipline and/or a professional practice?**

The final revised definition supports the view that clinical pharmacy encompasses both a scientific discipline and a professional practice. It avoids the potentially misleading distinction made in the initial draft of the definition between “science” and “practice” (since one informs the other), but instead states that clinical pharmacy aims to achieve its goals via “research” and “practice”.

**2. What are the aims of clinical pharmacy?**

The revised definition reflects the view that clinical pharmacy primarily aims to optimise the achievement of health-related goals in ways that support human dignity and personal choice (“person-centered goals”). The focus is on optimising the utilisation of medicines and implies that its goals are to be achieved via maximising medication effectiveness and safety. Person-centered goals aim to acknowledge patient preferences even when they might be at odds with evidence based practice. Public health goals, i.e. those that benefit society as a whole, can for example be achieved through prudent use of antibiotics to avoid microbial resistance. The revised definition avoids explicit mention of economic outcomes, which are considered of secondary importance to health-related goals. Although not explicitly mentioned by the revised definition (to reduce complexity), it is acknowledged that the achievement of person-centered goals may be constrained by public health goals and available resources.

**3. What does clinical pharmacy comprise?**

*Clinical pharmacy research.*

In order to further characterise clinical pharmacy research, several previous definitions have specified that clinical pharmacy is or draws on “natural science”, “biomedical science” or other disciplines, such as “clinical pharmacology” or “pharmaceutical technology” [6, 8, 10]. However, there is no internationally agreed taxonomy of scientific disciplines. Disciplines are constantly evolving, and new disciplines may emerge that align with the goals of clinical pharmacy research. The defining feature of clinical pharmacy research is therefore its aim. Research in clinical pharmacy may draw on disciplines as diverse as, but not necessarily restricted to, biomedical science (e.g. to study pharmacokinetics and pharmacodynamics), social pharmacy (e.g. studying how medicines are perceived, used, and managed by different actors in society), pharmacoepidemiology (e.g. to identify or quantify the benefits and harms of medicines), behavioural science (e.g. to understand and improve clinicians’ guideline adherence or patients’ adherence to medicine regimens), and health services research (e.g. to evaluate the impact of pharmaceutical care interventions). The focus of clinical pharmacy research is on optimising the utilization of existing medicines in the context of preventing and managing disease. This distinguishes it from other fields of scientific enquiry in pharmacy where the focus is on the discovery, pre-clinical testing and manufacturing of compounds and medicines (e.g. pharmaceutical biology, pharmaceutical chemistry, pharmaceutical technology or experimental pharmacology).

*Clinical pharmacy practice*

Given the evolving and dynamic nature of clinical pharmacy practice, the definition avoids listing specific services that may be provided under different labels in different settings. Clinical pharmacy practice is instead defined in terms of how its aims are achieved, namely via “cognitive (i.e. use of knowledge)”, “managerial (i.e. through development and delivery of services)” and “interpersonal (i.e. counselling)” tasks that may be required at any stage of the medication use process, namely (1) selection/design of medication regimens, (2) use/implementation of medication regimens, and (3) monitoring/adjustment of medication regimens by providers, individual patients and the public.

1. “Selection/design”—This encompasses decision-making around initiating (including advice on non-pharmacological treatment options), stopping or switching medicines as well as self-medication with non-prescription medicines. At the *individual patient* level, clinical pharmacy practitioners may draw on scientific or clinical evidence, and apply their knowledge and professional judgement to tailor the use of medicines to individual patient needs (e.g. defined by co-medication, co-morbidity, genetic profile, or psycho-social factors). At the *population level*, clinical pharmacy practitioners may target settings and prescribers (e.g. by developing/implementing guidelines as part of antibiotic stewardship or by implementing prescribing support software), or the public (e.g. by raising awareness and promoting the benefits of vaccination campaigns).
2. “Use/implementation”—This refers to the handling of medicines by patients and includes patient’s medication adherence. For example, clinical pharmacy practitioners may counsel patients or carers on the appropriate administration of medicines in patients with nasogastric tubes, or enable and motivate patients to take their medicines as agreed. However, it is important to note that clinical pharmacy practice is restricted to cognitive, managerial and interpersonal activities. The technical acts of compounding or physically administering pharmaceutical products does not fall within the scope of clinical pharmacy practice.
3. “Monitoring/adjustment”—This encompasses following-up on the effects of medicines by providers (e.g. via laboratory tests) often with the assistance of patients (e.g. via self-measurement of blood glucose) and adjusting treatment in response to the findings. In order to optimise monitoring and adjustment of medicines, clinical pharmacy practitioners may design and implement monitoring schedules and educate providers and/or patients on how and when to alter, stop or restart treatment, and also when to seek professional help.

*Philosophy of practice*

The term clinical pharmacy was created to formalise the contribution of pharmacists to patient care beyond their traditional roles, i.e. the development, manufacture and supply/dispensing of pharmaceutical products, and has supported a shift in focus from the medicine to understanding and addressing the needs of patients. Building on these developments, Hepler and Strand first argued that pharmacists should not only inform and educate, but also build therapeutic relationships with patients and assume responsibility for patient outcomes [14]. However, a recent survey suggests that many pharmacists are currently not willing to accept such responsibility [13]. Commonly cited reasons are unsupportive work environments (legal restrictions, lack of reimbursement for cognitive services and lack of access to clinical patient data), which imply that pharmacists’ opportunities to influence patient outcomes appear limited [15]. It is clear that pharmacists cannot be held responsible for actions or decisions of other professionals which are beyond their control. However, as professionals, clinical pharmacy practitioners are responsible for taking a proactive approach to the identification of medication problems and to do what is in their power to resolve them. Therefore, the philosophy of practice explicitly includes “care models in which pharmacists assume responsibility for achieving person-centered goals for individual patients as part of a multidisciplinary team” within the scope of clinical pharmacy practice.

**4. Who can provide clinical pharmacy (services)?**

A recent ESCP survey identified a lack of clarity as to whether health care professionals other than pharmacists could provide clinical pharmacy (services) [13]. While it is clear that non-pharmacy health care professionals can and do engage in activities that look to “optimise the utilisation of medicines” (e.g. medical doctors and clinical pharmacologists), we suggest that such practices are only termed as clinical pharmacy if they are conducted by pharmacists. Although this distinction limits the range of providers to pharmacists only, it has to be pointed out that such clinical pharmacy services will often be supported by other types of staff (e.g. pharmacy technicians or nurses obtaining medication histories or taking blood samples) or tools (such as decision support software). However, such supportive activities would not be considered clinical pharmacy in their own right.

**5. In which settings can clinical pharmacy services be provided?**

The revised definition explicitly states that clinical pharmacy practice may be conducted regardless of the setting. The word “clinical” therefore refers to the focus or orientation of clinical pharmacy activities (i.e. patients rather than medicines) and not the setting in which they are provided.

**6. What is the relationship between clinical pharmacy and pharmaceutical care?**

The PCNE has recently defined pharmaceutical care as “The pharmacist’s contribution to the care of individuals in order to optimise medicines use and improve health outcomes” [3]. However, the revised ESCP definition of clinical pharmacy avoids an explicit reference to pharmaceutical care, since definitions of pharmaceutical care may evolve independent of clinical pharmacy. In addition, the PCNE definition omits notions of responsibility in contrast to the original definition by Hepler and Strand: “Pharmaceutical care is the direct, responsible provision of medication-related care for the purpose of achieving definite outcomes that improve a patient’s quality of life” [14]. In contrast, the revised ESCP definition (Fig. 1) of clinical pharmacy explicitly incorporates this notion of responsibility as explained in more detail in Sect. 3.

Our definition of clinical pharmacy reflects the view that pharmaceutical care, both in its original definition [14] and its revised definition by PCNE [3], is a subset of clinical pharmacy practice. In contrast to pharmaceutical care, clinical pharmacy practice is not limited to activities that are directly targeted at individual patients, but also includes activities targeted at prescribers and/or the public, such as antibiotic stewardship, formulary and guideline development, safe electronic prescribing, and design of medicines management processes. In addition, clinical pharmacy is not only a professional practice but also a research discipline, whereas both of the above cited definitions frame pharmaceutical care as a practice rather than a field of scientific enquiry. Nevertheless, it is clear that pharmaceutical care will be equally informed by research and the outcomes from pharmaceutical care practice are evaluated and optimized through research [3]. Figure 1 illustrates that clinical pharmacy practice and by implication, pharmaceutical care as a subset of clinical pharmacy practice, is informed by knowledge generated through clinical pharmacy research as one among other relevant research disciplines. Vice versa, clinical pharmacy practice may inform clinical pharmacy research by generating relevant research questions.

## Discussion

We are presenting an updated revised definition and accompanying rationale for the term clinical pharmacy and its relationship to pharmaceutical care, developed through an iterative process with feedback from ESCP members, which focused on resolving six ambiguities around the term that were previously identified in a survey conducted by ESCP in 2014 [13]. Despite that the understanding of the term clinical pharmacy may have evolved since that time, the need for the consultation exercise was justified by the finding that the first circulated draft of the definition left up to 41% of participants unclear regarding the six questions posed. Although the participation rate in the consultation round (33.8%) may be considered relatively low in the context of confirmatory studies, the survey findings enabled the identification of key ambiguities and the 185 free text comments obtained from 65 (73%) participants provided a rich source of information for a qualitative exploration of views and opinions, as well as an opportunity to optimise the definition of the term. We took the opportunity to carefully consider each comment, of which the vast majority was judged to have been addressed (72%) or partially addressed (14%) in the second version of the definition (see supplementary electronic material S2).

Although many definitions of clinical pharmacy existed prior to this work, we were unable to source any that provided clarity on all of the six questions we looked to address [4–12]. For example, among an illustrative collection of 12 definitions from Europe and the United States, none included an explicit statement on the settings in which clinical pharmacy can be provided [13]. Most (but not all) limited clinical pharmacy to a professional practice, few named possible providers or addressed the relationship between clinical pharmacy and pharmaceutical care, only a minority specified outcomes targeted by clinical pharmacy practice, and none specified the aims of clinical pharmacy research. In contrast, the definition proposed here explicitly states that clinical pharmacy can be practiced by pharmacists regardless of setting, that it is both a professional practice and a field of research, and that (as a practice) it aims to achieve person-centered and public health goals. Uniquely, the ESCP definition defines the aims of clinical pharmacy research, i.e. to inform clinical practice including decision-making, health care organisation and/or policy, and thereby characterizes it as a discipline that combines clinical and health services research. In addition, the rationale clarifies that clinical pharmacy encompasses pharmaceutical care but is not limited to it.

## Conclusion

With the definition of clinical pharmacy presented here, we aim to resolve previous ambiguities around the term and to emphasize the features that distinguish it from more traditional fields of pharmacy practice and research. As such, the revised ESCP definition has the potential to support the wider implementation of clinical pharmacy practice and to further position clinical pharmacy as a field of scientific enquiry with a distinct research agenda. Future work could include an exploration of the perceived clarity and extent of agreement of the views expressed in this revised definition and its rationale, both within ESCP and beyond.

## Supplementary Information

Below is the link to the electronic supplementary material.

Supplementary file1 (DOCX 155 kb)

Supplementary file2 (DOCX 78 kb)
