# Supplementary material for: European Society of Clinical Pharmacy definition of the term clinical pharmacy and its relationship to pharmaceutical care: a position paper
Source: Int J Clin Pharm. 2022 Jun 6;44(4):837–42. doi: 10.1007/s11096-022-01422-7 (PMC9393137; doi:10.1007/s11096-022-01422-7)
Supplement: Supplementary file 1 — Supplementary file1 (DOCX 155 kb) [file 11096_2022_1422_MOESM1_ESM.docx]

Supplementary electronic material S1: Online Survey

**Consultation of ESCP members: Position paper on "What is Clinical Pharmacy? & What is its relationship to Pharmaceutical Care?**

**Background**

A series of definitions of Clinical Pharmacy have emerged since the early 1960s. We have previously argued that in order to gain support for an internationally harmonized definition of the term, differences in opinion regarding the term should be considered. In order to elicit such opinions, we have conducted a survey of 263 pharmacists affiliated with ESCP, which comprised questions regarding: (1) whether the term Clinical Pharmacy referred to a professional practice and/or a scientific discipline, (2) what professional activities it encompassed, (3) who could provide them, (4) in which setting and (5) to which end, and (6) whether and how it differed from Pharmaceutical Care. The findings were published in IJCP in 2016 [available at <https://www.ncbi.nlm.nih.gov/pubmed/27817173>].

Taking into account participants' responses, we have now drafted a position paper presenting a possible ESCP definition of Clinical Pharmacy and its rationale, which you find as attachments to the invitation email. The aim of this survey is to invite you as an ESCP member to comment on the definition or its rationale, before disseminating it more widely with the ultimate aim of achieving consensus on an internationally harmonised definition of Clinical Pharmacy.

**Proposed definition**

*Core definition:*Clinical pharmacy is a scientific discipline and a branch of pharmacy practice, which aims to optimise the therapeutic use of medicines by patients and professionals in order to maximise the likelihood that an optimal balance of clinical, humanistic and economic outcomes is achieved.

*Extension:*As an academic discipline, Clinical Pharmacy aims to generate and disseminate knowledge that directly informs clinical decision-making, health care organisation or policy in relation to the therapeutic use of medicines. As a professional practice, Clinical Pharmacy comprises services delivered by registered pharmacists or pharmaceutical staff acting under their responsibility, which target the therapeutic use of medicines at population and/or individual patient levels. Clinical Pharmacy services can be delivered in any setting. Where Clinical Pharmacy services target the therapeutic use of medicines in individual patients, Clinical Pharmacy practice and Pharmaceutical Care delivery (defined by the Pharmaceutical Care Network Europe as "The pharmacist’s contribution to the care of individuals in order to optimise medicines use and improve health outcomes") are synonymous.

**Survey instructions**

The survey comprises 17 questions. Questions 1 to 6 ask you about the clarity of the definition, whereas questions 7 to 12 ask you about your agreement with the definition. Please, answer questions 1 to 6 before reading the rationale that you find attached to the invitation email (the attachment can be found at the end of this Appendix document), and questions 7 to 12 after having read it. Question 13 allows you to provide any further comments on the definition and questions 14 to 16 ask you for some basic demographic information to allow us to characterise the sample of ESCP members participating in the survey. If you are happy for ESCP to contact you regarding this survey, please provide your contact details under item 17.

**Thank you very much for taking the time to help us with this important work!**

*** 1. Please, consider the proposed definition (including the extension) without the rationale: Does the definition provide a clear answer to the question as to whether the term Clinical Pharmacy refers to a professional practice and/or a scientific discipline?**

YES

UNCERTAIN

NO

If "NO" or "UNCERTAIN", please explain why and/or suggest an improvement:

**Question Title**

*** 2. Please, consider the proposed definition (including the extension) without the rationale: Does the definition provide a clear answer to the question as to which professional activities Clinical Pharmacy encompasses?**

YES

UNCERTAIN

NO

If "NO" or "UNCERTAIN", please explain why and/or suggest an improvement:

**Question Title**

*** 3. Please, consider the proposed definition (including the extension) without the rationale: Does the definition provide a clear answer to the question as to who can provide Clinical Pharmacy services?**

YES

UNCERTAIN

NO

If "NO" or "UNCERTAIN", please explain why and/or suggest an improvement:

**Question Title**

*** 4. Please, consider the proposed definition (including the extension) without the rationale: Does the definition provide a clear answer to the question in which settings Clinical Pharmacy services can be provided?**

YES

UNCERTAIN

NO

If "NO" or "UNCERTAIN", please explain why and/or suggest an improvement:

**Question Title**

*** 5. Please, consider the proposed definition (including the extension) without the rationale: Does the definition provide a clear answer to the question which outcomes Clinical Pharmacy aims to achieve?**

YES

UNCERTAIN

NO

If "NO" or "UNCERTAIN", please explain why and/or suggest an improvement:

**Question Title**

*** 6. Please, consider the proposed definition (including the extension) without the rationale: Does the definition provide a clear answer to the question whether and how the term Clinical Pharmacy differs from the term Pharmaceutical Care?**

YES

UNCERTAIN

NO

If "NO" or "UNCERTAIN", please explain why and/or suggest an improvement:

**Question Title**

*** 7. After reading the rationale for the definition:  Do you agree with the view expressed on whether Clinical Pharmacy refers to a professional practice, a scientific discipline, or both?**

YES

UNCERTAIN

NO

If "NO" or "UNCERTAIN", please explain why and/or suggest an improvement:

**Question Title**

*** 8. After reading the rationale for the definition:  Do you agree with the view expressed on which professional activities Clinical Pharmacy encompasses?**

YES

UNCERTAIN

NO

If "NO" or "UNCERTAIN", please explain why and/or suggest an improvement:

**Question Title**

*** 9. After reading the rationale for the definition:  Do you agree with the view expressed on who can provide Clinical Pharmacy services?**

YES

UNCERTAIN

NO

If "NO" or "UNCERTAIN", please explain why and/or suggest an improvement:

**Question Title**

*** 10. After reading the rationale for the definition:  Do you agree with the view expressed on the question in which settings Clinical Pharmacy services can be provided?**

YES

UNCERTAIN

NO

If "NO" or "UNCERTAIN", please explain why and/or suggest an improvement:

**Question Title**

*** 11. After reading the rationale for the definition:  Do you agree with the view expressed on which outcomes Clinical Pharmacy aims to achieve?**

YES

UNCERTAIN

NO

If "NO" or "UNCERTAIN", please explain why and/or suggest an improvement:

**Question Title**

*** 12. After reading the rationale for the definition:  Do you agree with the view expressed in the definition on whether and how Clinical Pharmacy differs from Pharmaceutical Care?**

YES

UNCERTAIN

NO

If "NO" or "UNCERTAIN", please explain why and/or suggest an improvement:

**Question Title**

**13. Do you have any other comments or suggestions regarding the definition (including the extension) or the rationale that you would like to share?**

**Question Title**

*** 14. What is your country of residence?**

**Question Title**

*** 15. When have you obtained your pharmacy degree?**

                                                                                                                                                                                 

**Question Title**

*** 16. Which setting(s) do you currently work in?**

Hospital

Community

University

Government organisation

Professional organisation

Other

**Question Title**

**17. If you are happy to be contacted by ESCP to discuss your responses to the survey, please provide your contact details below**

Name 

Email Address

Email attachment to the online survey

**European Society of Clinical Pharmacy definition of the term Clinical Pharmacy and its relationship to Pharmaceutical Care: Rationale (first draft)**

Background

A series of definitions of Clinical Pharmacy have emerged since the early 1960s. We have previously highlighted areas of uncertainty regarding what the term Clinical Pharmacy encompasses including its relationship to the related term Pharmaceutical Care and argued that in order to gain support for a harmonized definition, differences in opinion regarding the term should be considered.[^1^](#_ENREF_1) In order to elicit such opinions, we have conducted a survey of 263 pharmacists affiliated with ESCP [^1^](#_ENREF_1), which comprised questions that a panel of experts agreed to be relevant to informing a harmonised definition of Clinical Pharmacy or Pharmaceutical Care, namely: (1) whether the term Clinical Pharmacy referred to a professional practice and/or a scientific discipline, (2) what professional activities it encompassed, (3) who could provide them, (4) in which setting and (5) to which end, and (6) whether and how it differed from Pharmaceutical Care. The aims of this position paper are to provide a definition of Clinical Pharmacy that provides answers to all of the aforementioned questions and to explain the rationale for our stance on them.

ESCP definition of Clinical Pharmacy

**Core definition:** Clinical pharmacy is a scientific discipline and a branch of pharmacy practice, which aims to optimise the therapeutic use of medicines by patients and professionals in order to increase the likelihood that an optimal balance of clinical, humanistic and economic outcomes are achieved.

**Extension:** As an academic discipline, Clinical Pharmacy aims to generate and disseminate knowledge that directly informs clinical decision-making, health care organisation or policy in relation to the therapeutic use of medicines. As a professional practice, it comprises activities by pharmacy professionals targeting the use of medicines at population and individual patient levels. Where activities are targeted directly at individual patients, Clinical Pharmacy practice encompasses the delivery of Pharmaceutical Care (defined as the “pharmacist’s contribution to the care of individuals in order to optimise medicines use and improve health outcomes “[^2^](#_ENREF_2)).

Rationale

1. **Does the term Clinical Pharmacy refer to a scientific discipline and/or a professional practice?**

Consistent with strong agreement among participants in a previous ESCP survey[^1^](#_ENREF_1), the proposed definition supports the view that Clinical Pharmacy encompasses both a scientific discipline (94.2% agreement among survey participants) and a professional practice (93.9% agreement among survey participants). In addition, the term “pharmacy practice” is to be interpreted as not only encompassing a set of activities, but also the professional values and principles underpinning them. These include pharmacists assuming responsibility as members of multidisciplinary teams for attaining achievable drug therapy outcomes in line with individual patients’ preferences.

Several previous definitions have further characterised Clinical Pharmacy as being or drawing on “natural science” [^3^](#_ENREF_3), “biomedical [science]” [^4^](#_ENREF_4) or other disciplines, such as “clinical pharmacology” or “pharmaceutical technology”[^5^](#_ENREF_5). Given that there is no universally used taxonomy of scientific disciplines, and that disciplines are constantly evolving, the ESCP defines Clinical Pharmacy as a scientific discipline in terms of its aims, i.e. *to generate and disseminate knowledge that directly informs clinical decision-making, health care organisation or policy in relation to the therapeutic use of medicines*. The implication is that scientific enquiry in Clinical Pharmacy may draw on disciplines as diverse as (but not necessarily restricted to) behavioural science (e.g. to understand and improve patient adherence), biomedical science (e.g. to understand the relationship between pharmacokinetics and pharmacodynamics and guide dosing), clinical or population health science (e.g. to quantify the benefits and harms of treatments), and economic science (e.g. to evaluate the cost-effectiveness of interventions to inform funding decisions). The definition distinguishes Clinical Pharmacy as a scientific discipline from scientific enquiry into the physical, chemical or biological properties of drug products as opposed to their therapeutic use.

1. **What are the aims of Clinical Pharmacy?**

Consistent with findings of the ESCP survey, the ESCP definition supports the view that Clinical Pharmacy aims to optimise clinical outcomes (i.e. medication safety [95.4% agreement among survey participants] and effectiveness [95.1% agreement]), as well as humanistic (patient-centeredness [86.0% agreement among survey participants]) and economic (cost-effectiveness [83.5% agreement]) outcomes. The phrase “optimal balance” acknowledges that these outcomes may be mutually conflicting.

Previous definitions have described the aims of Clinical Pharmacy in terms of improving the medication use process, its outcomes or both, with few definitions specifying the outcomes targeted. The proposed definition emphasises the importance of process as an important intermediate goal that is largely under professional control and acknowledges that an optimised process does not guarantee optimised drug therapy outcomes.

1. **Which professional activities fall under the term Clinical Pharmacy?**

The ESCP definition states that Clinical pharmacy practice encompasses activities that may target individual patients or health care professionals (and the patient populations they serve). This is consistent with the ESCP survey finding agreement or strong agreement among participants that the term Clinical Pharmacy accommodated activities, such as ‘drug therapy optimisation at patient level (93.2% agreement)’, ‘treatment individualisation (93.9%)’, ‘informative patient counselling (87.1% agreement)’ and ‘compassionate patient counselling (81.0% agreement)’ as well as ‘drug therapy optimisation at provider level (87.5% agreement)’. The ESCP survey did however reveal more diverging opinions regarding the following activities: ‘compounding (71.1% disagreed that this constituted Clinical Pharmacy)’, ‘drug logistics (60.5% disagreed)’, ‘filling a prescription/ dispensing (54.8% disagreed)’, ‘drug administration (42.6% disagreed)’ and ‘public health promotion (43.5% disagreed)’. The ESCP definition limits the nature of Clinical Pharmacy activities to those that aim to optimise the therapeutic use of medicines by patients and professionals. Consistent with the majority of survey participants, activities solely relating to the production or distribution of medicines are not part of Clinical Pharmacy practice. The technical acts of dispensing and drug administration, as well as public health promotion (even when provided by pharmacy professionals) are not in themselves considered part of Clinical Pharmacy practice. Nevertheless, these activities often are accompanied by Clinical Pharmacy activities, such as providing information or counselling patients on the use of medicines or its effects.

1. **Who can provide clinical pharmacy (services)?**

The ESCP survey found agreement among participants that pharmacists could provide Clinical Pharmacy services (97%), and that informal carers (e.g. relatives) could *not* provide such services (93.1%), but there was uncertainty whether ‘other health care professionals’ could (74.8% of participants disagreed with this). Consistent with the majority of survey participants, the ESCP definition limits the range of providers to pharmacy professionals, including pharmacists or staff acting under their direction or on their behalf. Although it is acknowledged that non-pharmacy health care professionals can and do engage in activities that aim to “optimise the therapeutic use of medicines”, it is proposed that such services are inconsistent with the term Clinical *Pharmacy*. It is important to note, that the definition places no restrictions on who conducts Clinical Pharmacy research or disseminates knowledge relevant to Clinical Pharmacy practice.

1. **In which settings can Clinical Pharmacy services be provided?**

The ESCP survey found that there was strong agreement among participants that Clinical Pharmacy services could be provided in a ‘Hospital Ward or outpatient clinic (96.5%)’ and in a ‘Hospital pharmacy (92.7%)’. However, more uncertainty was reported regarding non-hospital settings (a ‘physician’s practice’ was not considered a site for provision of clinical pharmacy services by 24.1%, ‘community pharmacy’ by 29.3%, a ‘patient’s home’ by 31.7%, and ‘any other private or public space’ by 45.6% of participants, respectively). The ESCP definition does not specify the setting in which clinical pharmacy services should be provided, reflecting the view that such services may be provided irrespective of setting. The word “Clinical” therefore refers to the nature of Clinical Pharmacy activities rather than the setting in which they are provided.

1. **What is the relationship between Clinical Pharmacy and Pharmaceutical Care?**

The ESCP survey found that a majority of participants (76.0%) provided answers consistent with Clinical Pharmacy and Pharmaceutical Care partially overlapping but with both also having distinct characteristics. In contrast, the ESCP definition implies that Pharmaceutical Care is seen as part of Clinical Pharmacy practice, but that Clinical Pharmacy practice encompasses additional elements as shown in figure 1. The view expressed here is consistent with the current PCNE definition (“the pharmacist’s contribution to the care of individuals in order to optimise medicines use and improve health outcomes” [^2^](#_ENREF_2)), which restricts Pharmaceutical Care to activities by pharmacists targeting individual patients, and the view that Clinical Pharmacy additionally encompasses activities targeting health care professionals or populations. Examples of the latter are the development of policies or guidelines about the therapeutic use of medicines or medicines information services. Figure 1 also illustrates that the term Clinical Pharmacy encompasses both a scientific discipline and a health care practice, the latter being informed by the former. It is important to note that the benefits of Clinical Pharmacy research are not restricted the pharmacy profession.

**Figure 1:** Relationship between Clinical Pharmacy science and practice and Pharmaceutical Care


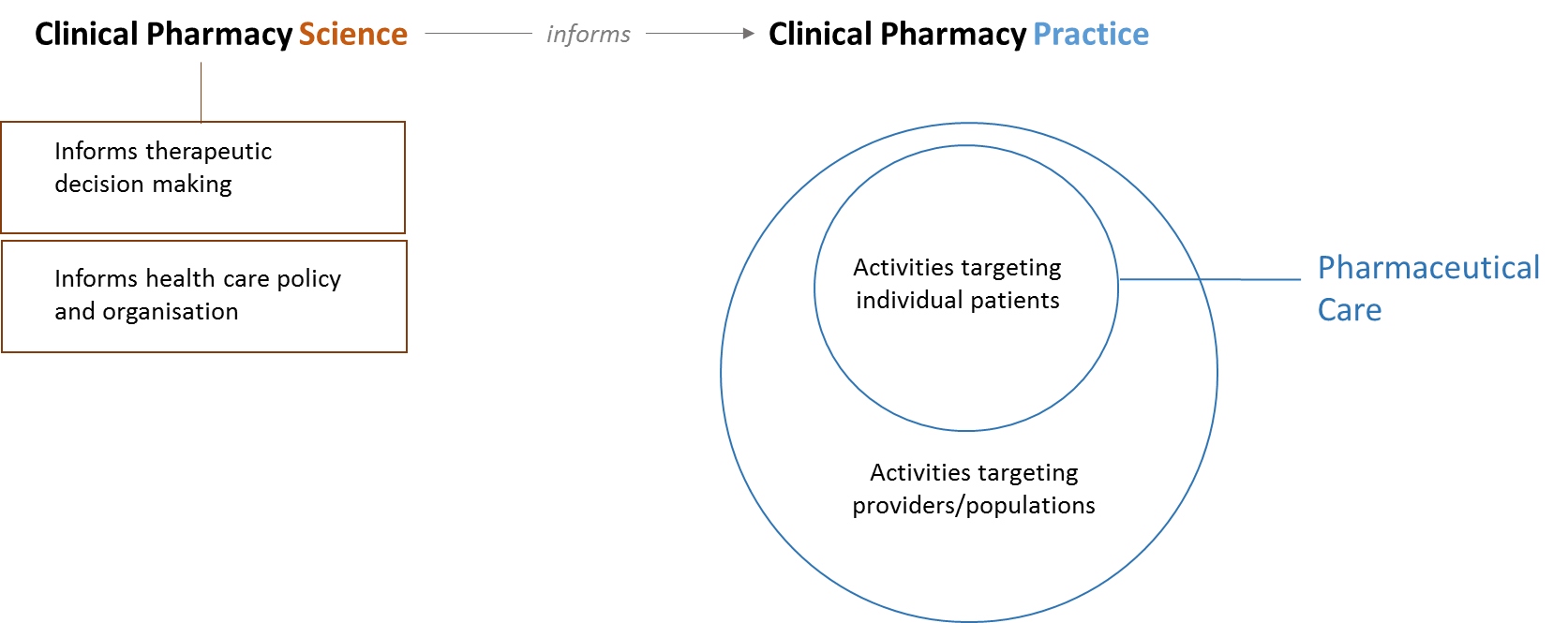


Summary

To summarize, there are national and regional differences throughout Europe as to how the term Clinical Pharmacy is used and understood. Nevertheless, the proposed definition is consistent with the opinions of the majority of participants in a previous ESCP survey with regards to (1) the term Clinical Pharmacy referring to both a professional practice and a scientific discipline, (2) the remit of Clinical Pharmacy practice being limited to those that aim to optimise the therapeutic use of medicines by patients and professionals, (3) providers of Clinical pharmacy services being limited to pharmacists or their staff, (4) the provision of such services not being limited to a particular setting and (5) the desired outcomes of Clinical Pharmacy practice comprising financial, economic and humanistic outcomes. Although the relationship between Clinical Pharmacy and Pharmaceutical Care proposed here appears to differ from the majority of participants in the previous ESCP survey, it is consistent with the recently updated definition of Pharmaceutical Care by the Pharmaceutical Care Network Europe. We hope that the extensive work behind the current ESCP definition will facilitate international consensus on a harmonised definition of term Clinical Pharmacy and its relationship to Pharmaceutical Care.

References

1. Dreischulte T, Fernandez-Llimos F. Current perceptions of the term Clinical Pharmacy and its relationship to Pharmaceutical Care: a survey of members of the European Society of Clinical Pharmacy. *International Journal of Clinical Pharmacy* 2016;38(6):1445-56.

2. Allemann SS, van Mil JW, Botermann L, et al. Pharmaceutical care: the PCNE definition 2013. *International Journal of Clinical Pharmacy* 2014;36(3):544-55.

3. Bundesvereinigung Deutscher Apothekerverbaende (ABDA) und Deutsche Pharmazeutische Gesellschaft (DPhG). Was kann Klinische Pharmazie, und für wen ist sie? *Pharm Ztg* 1998;143(1962):1210.

4. Calop J AB, Brudieu. Definition de la pharmacie clinique. In: Gimenez F CJ, Limat S, Fernandez C, ed. Pharmacie Clinique et Therapeutique. Paris: Elsevier-Masson 2008.

5. Società Italiana di Farmacia Clinica (Italian Society of Hospital Pharmacy). Available at <http://www.sifac.it/node/149> [last accessed 06/12/2015].
