# Supplementary material for: European Society of Clinical Pharmacy definition of the term clinical pharmacy and its relationship to pharmaceutical care: a position paper
Source: Int J Clin Pharm. 2022 Jun 6;44(4):837–42. doi: 10.1007/s11096-022-01422-7 (PMC9393137; doi:10.1007/s11096-022-01422-7)
Supplement: Supplementary file 2 — Supplementary file2 (DOCX 78 kb) [file 11096_2022_1422_MOESM2_ESM.docx]

| Supplementary electronic material S2: Responses to free text comments |
| --- |

| **Free text comments** | **Authors’ consensus assessment of participant responses** | |
| --- | --- | --- |
|  | Resolved by  revised  definition? | Comment |
| 1. **Does Clinical Pharmacy refer to a scientific discipline and/or a professional practice?** | | |
| It would be helpful to have two different terms on clinical pharmacy as a science and as pharmacy practice. | Yes | The extension of the definition differentiates between CP practice and research. |
| Too long, too many 'side-lines' I would prefer a shorter statement. | Yes | The definition and extension were shortened with reduced complexity. |
| Yes, it is very clear, although in my opinion scientific discipline should not be mentioned seperately. Every pharmacy discipline should be scientifically. | Yes | “Scientific discipline” is no longer mentioned in the revised definition. The scientific aspect of CP is reflected by the word “research”, which we consider a less ambiguous and more readily understood term. |
| In the extension, I would not introduce the term "Academic discipline", but stick to "Scientific discipline" as these terms are the same to me. | Yes | Neither “Academic discipline” nor “Scientific discipline” are mentioned in the revised definition. The scientific aspect of CP is reflected by the word “research”, which we consider a les ambiguous and more readily understood term. |
| I agree but I do not think that it needs to be seperated in this strong way, as it is obvious that any pharmaceutical activity is based on science. | Yes | “Scientific discipline” is no longer mentioned in the revised definition. The scientific aspect of CP is reflected by the word “research”, which we consider a less ambiguous and more readily understood term. |
| "CP is a scientific discipline and professional practice within the field of pharmacy practice, which..." You may want to cut out the pharmacy practice part in the core definition and just elaborate on it in the extension. | Yes | “Pharmacy practice” is not mentioned in the revised definition. |
| Could be 'Clinical pharmacy is a scientific branch of pharmacy practice. | No | We consider all pharmacy practice to be informed by science/research and therefore would want to avoid the impression there is a scientific and (by implication) a non-scientific branch. |
| What is the difference between an academic and a scientist? | Yes | Neither “Academic discipline” nor “Scientific discipline” are mentioned in the revised definition. |
| It should be clarify the term 'pharmacy practice'. | Yes | In the revised definition, we no longer refer to “pharmacy practice” thereby avoiding a term that requires defining in its own right. |
| While clinical pharmacy has a scientific component, it also has a clinical component. Therefore, it is not a purely scientific discipline. | Yes | “Scientific discipline” is no longer mentioned in the revised definition. We differentiate between research and practice. In the revised definition we clarify that CP research informs CP practice. In the rationale we state that “CP practitioners may draw on scientific or clinical evidence and apply their knowledge and professional judgement to tailor the use of medicines to individual patient needs”. |
| In the extension, clinical pharmacy is referred to as an academic discipline, lending a third facet to the overall discipline. | Yes | Neither “Academic discipline” nor “Scientific discipline” are mentioned in the revised definition or rationale. |
| It seems that the term "scientific" as a modifier is not fully descriptive of the discipline of clinical pharmacy. I would suggest omitting the word "scientific" and simply stating that "Clinical pharmacy is a discipline. | Yes | Neither “Academic discipline” nor “Scientific discipline” are mentioned in the revised definition or rationale. |
| There are elements in the definition with unclear content, e.g the not defined "clinical" which in my opion would be improved using "patient-centred" or "-oriented" and in contrast to the drug/health care product perspective. | No | While we agree that “patient-centered or -oriented” would avoid the ambiguity of “clinical” (which may be interpreted as referring to a setting rather than its focus), our aim here was to define Clinical Pharmacy. |
| This would also allow to eliminate the term "humanistic" which again is not self-explaining. | Yes | The revised definition avoids the term “humanistic” outcomes, which refers to the ECHO model of pharmacoeconomic research (Kozma CM, Reeder CE, Schulz RM. Economic, clinical, and humanistic outcomes: a planning model for pharmacoeconomic research. Clin Ther. 1993 Nov-Dec;15(6):1121-32; discussion 1120. PMID: 8111809). Instead, the desired outcomes of CP are characterized by the term “patient-centered goals” which are intended to encompass any goals that are deemed important by the patient. |
| The term "scientific" is also not used in a clear and commonly understood way like based on scientific principles. This also shows the problem e.g. of a master title "clinical pharmacy" and "pharmaceutical sciences". | Yes | “Scientific discipline” is no longer mentioned in the revised definition. |
| 1. **What does Clinical Pharmacy (research and practice) comprise?** | | |
| *Research* |  |  |
| Does not provide clarity of the sciences that are applied. | Yes | The revised rationale now states that “there is no internationally agreed taxonomy of scientific disciplines. Disciplines are constantly evolving, and new disciplines may emerge that serve the goals of CP. The defining feature of CP research is therefore its aim. Research in CP may draw on disciplines as diverse as (but not necessarily restricted to) biomedical science (e.g. to understand the relationship between pharmacokinetics and pharmacodynamics to guide dosing), pharmacoepidemiology and pharmacoeconomics (e.g. to quantify the benefits and harms of drugs or drug regimens relative to their cost via observational or experimental approaches), behavioural science (e.g. to understand and improve clinicians’ adherence to a guideline or patients’ adherence to agreed drug regimens), and health services research (e.g. to evaluate the impact of pharmaceutical care interventions on health care processes and outcomes)”. |
| The rationale should include the term pharmacotherapy rather than natural science, biomedical science, etc. or be added to them. | Yes | The rationale lists these fields of research as examples of what other definitions have used, rather than stating that CP is limited to these disciplines. The revised rationale argues that because “there is no internationally agreed taxonomy of scientific disciplines” and because “disciplines are constantly evolving, and new disciplines may emerge that serve the goals of CP” that “ the defining feature of CP research is therefore its aim”. |
| I don't see how the current definition differentiates it from other branches of the profession which are optimising medicines use by actions at a population level, individual clinician i.e. education. | Yes | The revised rationale now makes that distinction explicit by stating that “The focus of CP research is on optimising the utilization of existing drug products in the context of preventing and managing disease, which distinguishes it from other fields of scientific enquiry in pharmacy that focus on the discovery, pre-clinical testing and manufacturing of compounds and drug products (e.g. pharmaceutical biology, pharmaceutical chemistry, pharmaceutical technology or experimental pharmacology)“. |
| *Practice activities* |  |  |
| A professional practice is by definition related to a complex knowledge domain. See definitions of professions and professional practice. A professional practice implies a complex knowledge domain. However, I would prefer two different terms to distinguish that more clearly. | Yes | The revised definition and rationale include both terms (knowledge and practice) and clarify their relationship by stating that “CP research generates knowledge which informs practice”. |
| Why only cognitive tasks? Also social/communicational tasks. | Yes | The revised definition now states that “Clinical Pharmacy practice comprises cognitive, managerial and counselling activities”. |
| In the extension, "target the therapeutic use of medicines at population and/or individual patient levels" does not make it clear that services can be delivered to other Health care professionals. I suggest to write "target the therapeutic use of medicines at population, PROVIDER, and/or individual patient levels". | Yes | The revised definition clarifies that CP practice targets the appropriate selection, administration and monitoring of medicines by providers and patients. |
| See remark before about individual compounding. | Yes | The revised rationale clarifies that “The technical acts of compounding or physically administering pharmaceutical products does not fall within the scope of CP practice”. |
| I feel it missed some parts not only around therapeutic use of medicines but also 1-herbs and vitamins,  licensed, unlicensed and off-label 2-therapeutic as well as prevention 3-counselling regarding other aspects -not only about medicines- such as about lifestyle modification. | Partly/  unclear | The revised rationale clarifies that CP practice also targets decision making around initiating (including advice to prioritise life style modification or non-pharmacological treatment options), stopping (“deprescribing”) or switching medicines as well as self-medication (i.e. selection by patients) with non-prescription medicines. |
| Limited information stated about professional activities. | Partly/  unclear | The revised rationale has been expanded to describe the scope of CP practice activities more comprehensively. It provides an expanded number of examples, but it is clear that it cannot be comprehensive in this regard. |
| The use of medicine by professionals does not reflect what professional activities this comprises. | Partly/  unclear | The revised rationale clarifies that “In order to optimise use and selection of medicines at population level, CP practitioners may target prescribers (e.g. by developing/implementing guidelines as part of antibiotic stewardship or by implementing prescribing support software), or the public (e.g. as part of health awareness campains).” The revised rationale has been expanded to describe the scope of CP practice activities more comprehensively. It provides an expanded number of examples, but it is clear that it cannot be comprehensive in this regard. |
| The activities are not mentioned concretely, therefore you could optimise the definition before ticking yes in this question. | Partly/  unclear | The revised rationale now provides an expanded number of examples, but it is clear that it cannot be comprehensive in this regard. |
| Too narrow. Antimicrobial stewartship, public health and policy not considered. | Yes | The revised rationale now provides an expanded number of examples, but it is clear that it cannot be comprehensive in this regard. It states that “In order to optimise use and selection of medicines at population level, CP practitioners may target prescribers (e.g. by developing/implementing guidelines as part of antibiotic stewardship or by implementing prescribing support software), or the public (e.g. as part of health awareness campains)”. |
| Is indiviual compounding also part of clinical pharmacy as it involves the production of a drug for therapeutic use? | Yes | The revised rationale clarifies that “The technical acts of compounding or physically administering pharmaceutical products does not fall within the scope of CP practice.” The term CP has been coined to differentiate it from traditional activities that focus on drug products rather than on optimising the medication use process. |
| A description of some typical activities would be helpful. For someone who is not in the field, this definition may seem very large and unclear. | Partly/  unclear | The revised rationale now provides an expanded number of examples, but it is clear that it cannot be comprehensive in this regard. |
| Because of too many sidelines it becomes less clear. | Yes | The revised definition has been shortened with reduced complexity |
| Does not define the activites that constitute Clinical Pharmacy. | Partly/  unclear | The revised rationale now provides an expanded number of examples, but it is clear that it cannot be comprehensive in this regard. |
| I think it does not specify activities but I would not expect that from a definition. The activities can be defined in a certain setting in accordance with the definition. | Partly/  unclear | We agree. The revised rationale now provides an expanded number of examples, but it is clear that it cannot be comprehensive in this regard. |
| I am a bit uncertain if the opimization of population based medication therapy and of the medication use process is considered enough. | Yes | The revised rationale now makes explicit reference to the medication use process It states that “CP practice is therefore defined in terms of how its aims are achieved, namely via “counselling”, “managerial” and “cognitive tasks” that may target any stage of the medication use process, namely “use/selection, administration and/or monitoring/adjustment of medicines by providers and/or patients”. In relation to population based medication therapy, it states that “In order to optimise use and selection of medicines at population level, CP practitioners may target prescribers (e.g. by developing/implementing guidelines as part of antibiotic stewardship or by implementing prescribing support software), or the public (e.g. as part of health awareness campains)”. |
| "Only" the objectives are mentioned. There is no clear answer how exactly one would achieve such goals. | Partly/  unclear | The revised definition specifies the “how” by stating “Clinical Pharmacy practice comprises cognitive, managerial and counselling activities by pharmacists regardless of setting, which target the appropriate selection, administration and monitoring of medicines by providers and patients. The revised rationale provides an expanded range of examples of professional activities targeting individual patients, providers and the public. |
| Where is the list? | Partly/  unclear | A definition cannot be expected to provide a comprehensive list of activities that fall under the scope of CP. In addition, CP is a dynamic and evolving field, which imples that any list of activities will be outdated soon after its release. The revised rationale now provides an expanded number of examples, but it is clear that it cannot be comprehensive in this regard. |
| I find the servies not very specific. | Partly/  unclear | A definition cannot be expected to provide a comprehensive list of activities that fall under the scope of CP. In addition, CP is a dynamic and evolving field, which imples that any list of activities will be outdated soon after its release. The revised rationale now provides an expanded number of examples, but it is clear that it cannot be comprehensive in this regard. |
| With regard to the number of competences a pharmacist has to have, this is too narrow. | Yes | The revised definition expands the scope of CP activities by managerial and counselling activities, and the recipients of CP activities by “the public”. |
| *Philosophy of practice* |  |  |
| The view expressed should refer to both, given the opportunity to define the philosophy of practice in an appropriate way to present the complete picture where philosophy of practice and delivery of comprehensive medication management services are twinned. | Partly/  unclear | The revised rationale includes a section on philosophy of practice. In order to avoid introducing further ambiguity/complexity, we decided against introducing additional terms, such as medication therapy management, which may themselves be defined/understood in various ways. |
| There are differences in describing practice/delivery of a service. In the evolution of PC we now define the philosophy of practice (a practice in which the practitioner takes the responsibility for a patient’s medicines-related needs, and is held accountable for this commitment) in other words being a reflective practitioner, and the delivery of comprehensive medication therapy management services (see Cipolle et a. 2004 and PCPCC Medication Management Task Force, 2012). | Yes | The notion of responsibility is included in the definition’s extension (“Clinical Pharmacy practice encompasses (but is not limited to) care models in which pharmacists assume responsibilty for achieving person-centered goals for individual patients as part of a multidisciplinary team.”) The rationale expands on this notion, acknowledging that pharmacists cannot be held responsible for actions of other professionals that are not under their control. |
| The consequence is that pharmacists cannot assume they have a unique responsibility for delivering it, now or in the future. Future societies have the possibility of receiving care in medicines use by various other means. For the profession to remain relevant in society it must focus on ensuring pharmacists are regarded as quality performers responding to patient needs. | Yes | The revised definition states that pharmacists “may assume responsibility as part of a multidisciplinary team”. The notion of responding to patient needs is captured in the definition by the stated aim “to achieve person-centered …. goals” which is expanded on in the rationale by stating that this aim is to be achieved “ in a way that respects human dignity and personal choice” In addition, the rationale states that “CP practitioners may draw on scientific or clinical evidence and apply their knowledge and professional judgement to tailor the use of medicines to individual patient needs”. |
| I find the sentence that the services can be delivered unsatisfying, bcause I feel the service should only be provided in settings where mnagement and funding guarantees that the pharmacist or a person s/he supervises is delivered independendently in the sense that it is not provided in e.g. managerial dependency. | Yes | The revised definition avoids the notion of “service” and instead uses the terms “practice”, “activities” and “models of care”. |
| The services and delivery model does not comply with the inherent professional practice model where complex professional knowledge is effectively shared with patients, professionals and society. (MacIntyre model of professional practice as a vitious practice). | Yes | The revised definition avoids the notion of “service” and instead uses the terms “practice”, “activities” and “models of care”. |
| A professional practice by definition aimes at balancing different values by the professional in practice. I suggest defining ‘profession’ and ‘professional practice' precedes the consensus proces within the profession about what the practice is about and how this can be described. | No | While it may be the case that there are also ambiguities around the nature of a profession and a professional practice in general, we do not consider resolving these questions a pre-requisite to defining CP practice. |
| Suggest adding responsibility for the outcomes. | Yes | The notion of responsibility is addressed in the definition in the phrase “care models in which pharmacists assume responsibility within a multidisciplinary team and expand on this in the rationale under “philosophy of practice”. |
| Again, need to add the accountability for the results or non results. | Yes | The notion of responsibility is addressed in the definition in the phrase “care models in which pharmacists assume responsibility within a multidisciplinary team and expand on this in the rationale under “philosophy of practice”. |
| Target therapeutic use does not say anything on medication safety / surveillance, accustoming treatment in relation to personal goals etc. | Yes | The revised definition now states that the aim of CP is to achieve “person-centered goals….” The revised rationale states that CP practice “may target any stage of the medication use process, namely “use/selection, administration and/or monitoring/adjustment of medicines”. It also states that CP’s “focus on optimising the utilisation of medicines implies that its goals are to be achieved via maximising medication effectiveness and safety.” The rationale states further that “CP practitioners may draw on scientific or clinical evidence and apply their knowledge and professional judgement to tailor the use of medicines to individual patient needs”. |
| 1. **What are the aims of Clinical Pharmacy?** |  |  |
| Must include Public Health. | Yes | The revised definition explicitly refers to public health goals: “Clinical pharmacy aims to optimise the utilisation of medicines through practice and research in order to achieve person-centered and public health goals”. |
| Including economic outcomes in the definition compromises the scientific and humanistic foundation of the praxis directly. | Yes | The revised definition avoids reference to economic outcomes and cost. The revised rationale clarifies that such outcomes are secondary to patient-centered outcomes:” CP primarily aims to optimise the achievement of health-related goals in a way that respects human dignity and personal choice (person-centered goals), as well as health-related goals that are considered important to society as a whole (public health goals), such as limiting microbial resistence. By implication, saving medication related cost is considered secondary to these aims. Nevertheless, achieving individual patient wishes may be constrained by societal goals and available resources. |
| See previous comment. The inclusion/exclusion of public health. | Yes | The revised definition explicitly refers to public health goals: “Clinical pharmacy aims to optimise the utilisation of medicines through practice and research in order to achieve person-centered and public health goals”. |
| I support 'humanistic outcomes' although they are nt so clear. I guss this relates to situations like terminal pain management and outcomes like trust of patients in pharmacists and their advice/services. I strongly support such outcomes. | Yes | The revised definition avoids the (in this context) not readily understood term “humanistic” but refers to person-centered goals instead. The rationale specifies that what is meant by this term is the “achievement of health-related goals in a way that respects human dignity and personal choice”. |
| Need to include improved patient outcomes. | Yes | In the revised definition, the outcomes CP aims to achieve are captured by the term “patient-centered goals”. We use the word “optimise” rather than improve, because it could be understood as CP correcting errors of others. The aim is that CP practice becomes an integral part of routine health care and as such contributes to the achievement of person-centered goals as part of a multidisciplinary team. |
| The societal responsibility on cost effectiveness is missing. | Yes | The revised definition clarifies that the primary aims of CP are achieving health related individual and public health goals and that saving cost is secondary to these aims. We share the concern of other survey participants that CP may be peceived as giving undue weight to saving cost, and we therefore avoid explicit reference to it in the definition. |
| The understanding of the term humanistic is not intuitive for me, even if its phylosophical definition reflects the intention. Welfare? Well-being? | Yes | The revised definition avoids the (in this context) not readily understood term humanistic. The rationale specifies that what is meant here is that “health related goals are to be achieved in a way that respects human dignity and personal choice”. |
| Thinks like the balance of make it less clear. | Yes | The revised definition now avoids this level of complexity. However, the revised rationale states that achieving person-centered goals may be constrained by public health goals and available resources. |
| Although it did, I believe it missed other outcomes (especially last sentence that hinted that clinical pharmacy only focuses on 'health outcomes' in the case of individual patients). | Yes | The revised definition now states that CP aims to achieve person-centered and public health goals. The rationale specifies that what is meant by this term is the “achievement of health-related goals in a way that respects human dignity and personal choice” Therefore, while the ultimate aim is to achieve “person-centered ... goals” in the broadest sense, the primary contribution of CP is on improving health by optimising the utilisation of medicines. |
| The aims and the target does not explain the kind of outcomes must be achieved. | Yes | The revised definition now states that CP aims to achieve person-centered and public health goals via optimising the utilization of medicines. The revised rationale specifies that CPs focus is on “maximising medication effectiveness and safety” and that this is to be achieved “in a way that respects human dignity and personal choice”. |
| Clinical Pharmacy is the framework by which pharmaceutical care is delivered. It also ecompassed medicines management and medicines optimisation Partlly adddressed, medication optimization might be added to CP practice. | Partly/  unclear | The figure illustrating the relationship between CP practice and research and PC is consisten with this notion. However, we avoid the use of other terms, such as medicines management and medicines optimization, which would require definitions in their own right in order to be readily understood. |
| At a broad level, yes; but "clinical, humanistic and economic" are quite broad so im not sure if that is a "clear answer to the question". | Yes | The revised definition now states that CP aims to achieve person-centered and public health goals via optimising the utilization of medicines. The revised rationale specifies that CPs focus is on “maximising medication effectiveness and safety” and that this is to be achieved “in a way that respects human dignity and personal choice”. The rationale states that economic goals are deemed to be of secondary importance to the achievement of health related goals and are therefore no lobger mentioned in the definition. |
| The term of "therapeutic" use might be limiting in some instances as it is when separating from some more basic services like ready-to-use preparation, logistics incl. distribution, labelling. There are important combined aspects to consider or told in a black and white fashion: drug therapy use would still work in Absence of clinical pharmacy but not the other way round. | Partly/  unclear | The revised definition extends the scope of CP practice by stating that “Clinical Pharmacy practice comprises cognitive, managerial and counselling activities by pharmacists regardless of setting, which target the appropriate selection, administration and monitoring of medicines by providers, individual patients and the public”. However, in order to distinguish itself from more traditional functions of pharmacy practice, it intentionally excludes activities focused on drug products, such as the technical acts of compounding, administration, and distribution/logistics. |
| I do not agree with the inclusion of economic outcomes. A priori, there should be a clear distinction of clinical pharmacy as a scientific discipline for outcomes of health and well-being versus pharmacoeconomics. I strongly advocate for taking economic outcomes out of the definition to keep scientific professional standards high and keep patients' trust that they get the best independent advice. I however could agree to discuss economical aspects with patients as part of pharmaceutical care, because it is caring to give the patient options, explaining that cheaper is as good or the cheaper, afordable option is better than nothing. If that is a good way forward, pharmaceutical care in figure 1 would have to clarify that it contains that additional economic advive. I could agree with that. Well formulated now. | Yes | The revised definition avoids reference to economic outcomes and cost. The revised rationale clarifies that such outcomes are secondary to patient-centered outcomes:”CP primarily aims to optimise the achievement of health-related goals in a way that respects human dignity and personal choice (person-centered goals), as well as health-related goals that are considered important to society as a whole (public health goals), such as limiting microbial resistence. By implication, saving medication related cost is considered secondary to these aims. Nevertheless, achieving individual patient wishes may be constrained by societal goals and available resources. |
| I propose not only to focus on the therapeutic outcome but also to avoid drug-related problems and improve patient safety (see also PCNE position paper regarding medication reviews). | Yes | The revised definition now states that CP aims to achieve person-centered and public health goals via optimising the utilization of medicines. The revised rationale specifies that CPs focus is on “maximising medication effectiveness and safety” and that this is to be achieved “in a way that respects human dignity and personal choice”. |
| Clinical and humanistic outcomes are aligned with the specific professional practice. Responsible Economic outcomes are reached by the professional by deliberation and weghing individual' patients values against socieatal values. | Yes | The revised definition clarifies that the primary aims of CP are achieving health related individual and public health goals and that saving cost is secondary to these aims. We share the concern of other survey participants that CP may be peceived as giving undue weight to saving cost, and we therefore avoid explicit reference to it in the definition. The revised rationale states that “… saving medication related cost is considered secondary to these aims. Nevertheless, achieving individual patient wishes may be constrained by societal goals and available resources”. |
| Optimise medicine use is quite broad. This implies personal goals, reduction of side effects, prevention, medication surveillance. It would help to name these sub outcomes! | Yes | The revised definition now states that CP aims to achieve person-centered and public health goals via optimising the utilization of medicines. The revised rationale specifies that CPs focus is on “maximising medication effectiveness and safety” and that this is to be achieved “in a way that respects human dignity and personal choice”. |
| Kind of outcomes are absent. | Yes | The revised definition now states that CP aims to achieve person-centered and public health goals via optimising the utilization of medicines. The revised rationale specifies that CPs focus is on “maximising medication effectiveness and safety” and that this is to be achieved “in a way that respects human dignity and personal choice”. The rationale also explains that economic outcomes are of secondary importance but that achieving person-centered goals may be constrained by public health goals and resources. |
| The public health, population outcomes are not considered e.g microbial resistance. | Yes | The revised definition now explicitly names public health as an outcome and the rationale lists antimicrobial resistance as an example. |
| Patient outcomes are defined in a limited way. Quality of practice/service provided as perceived by patients is not mentioned. | Yes | The revised definition now states that CP aims to achieve person-centered … goals and the revised rationale specifies that this is to be achieved “in a way that respects human dignity and personal choice”. |
| Three different types of outcomes are mentioned one of which (economic) is not aligned with the central scope of a professional practice and will give rise to internal conflicts and blurring of the professional scope of the practice. | Yes | The revised definition clarifies that the primary aims of CP are achieving health related individual and public health goals and that saving cost is secondary to these aims. We share the concern of this and other survey participants that CP may be peceived as giving undue weight to saving cost, and we therefore avoid explicit reference to it in the definition. The revised rationale states that “… saving medication related cost is considered secondary to these aims. Nevertheless, achieving individual patient wishes may be constrained by societal goals and available resources”. |
| 1. **Who can provide clinical pharmacy (services)?** |  |  |
| Change pharmaceutical to pharmacy. | Yes | The revised definition limits the range of CP providers to pharmacists. However, the revised rationale clarifies that CP practice may be supported by pharmacy or non-pharmacy staff. This notion is consistent with other professions. For example, the practice of medicine is limited to medical doctors, who may be supported by nurses or medical assistants. |
| Should be based on a competancy framework. | No | Given the wide range of CP activities, we consider it beyond the scope of this work to specify the competencies of CP practitioners. |
| In my experience, the core competencies of registered pharmacists are not always sufficient to meet the demands of clinical pharmacy. A post-graduate degree in clinical pharmacy is almost always required. Not for this definition. | No | Given the wide range of CP activities, the required qualifications will differ. In addition, pre-graduate education in pharmacy differs widely across Europe. Few phrmacists have access to formal postgraduate education in CP, and making this a pre-requisite to practice CP would be a large barrier to its implementation and spread to the disadvantage of patients. In the revised rationale, we therefore specifiy that pharmacists must be “appropriately qualified”, but consider it outside the scope of this work to specify exactly what this means. |
| A registred pharmacist might be understood in various ways in different countries and not cover the essential competencies on a European scale. Not adddressed, but also not for the definition. | No | In the revised rationale, we specifiy that pharmacists must be “appropriately qualified”, but consider it outside the scope of this work to specify exactly what this means. |
| The definition is clear, but I disagree that the task may be delegated to appropriately trained pharmaceutical staff, Furthermore, I think that the pharmacists providing clinical pharmacy should have extra training to obtain the skills needed for clinical pharmacy. | Partly/  unclear | In the revised rationale, we now avoid the term “delegate” and clarify that technical staff may support CP practitioners. This notion is consistent with other professions. For example, the practice of medicine is limited to medical doctors, who may be supported by nurses and technical assistants. We specifiy that pharmacists must be “appropriately qualified”, but consider it outside the scope of this work to specify exactly what this means. |
| Need to define "Pharmaceutical staff" - who does that involve other than pharmacists? Do you mean e.g. clinical pharmacologists? | Yes | The revised definition and rationale avoid the potentially ambiguous term pharmaceutical staff. CP providers are limited to pharmacists, who may be supported by other staff. |
| "Registered pharmacists or pharmaceutical staff" - only pharmacists? Who emcopasses 'pharmaceutical staff'? | Yes | The revised definition and rationale avoid the potentially ambiguous term pharmaceutical staff. CP providers are limited to pharmacists, who may be supported by other staff. |
| Pharmaceutical staff is very broad and could be misinterpreted. | Yes | The revised definition and rationale avoid the potentially ambiguous term pharmaceutical staff. CP providers are limited to pharmacists, who may be supported by other staff. |
| What is about physicians or nurses working under the responsability of a pharmacist? | Yes | The definition limits CP providers to pharmacists, who may be supported by other staff. This could include nurses and (theoretically) also physicians. |
| I think clinical pharmacy can also be performed by others than pharmaceutical staff (e.g. nurses) as long as it is done under supervision of a clinical pharmacist. | Yes | The revised definition limits CP providers to pharmacists, who may be supported by other staff. |
| Professionals are a wide definition for people doing clinical pharmacy. But how necessary is that? I would leave this out. | No | The revised definition limits CP providers to pharmacists, who may be supported by other staff. The above comments demonstrate that this an area of ongoing controversy. We therefore consider it useful to clarify this point. |
| The term "registered pharmacists", maybe can be substitued with "trained/graduaded/specialized pharmacists" to explain that the pharmacists is specialized in clinical pharmacy (maybe only in some countries). | Partly/  unclear | The term "registered pharmacists" is no longer used in the revised definition. We consider any further qualification of the term redundant in the definition (since the title pharmacist is reserved to those with a university qualification in pharmacy). In the revised rationale, we specifiy that pharmacists must be “appropriately qualified”, but consider it beyond the scope of this work to specify exactly what this means. |
| The definition did not specify who can provide it (for example if advanced training is needed or not). | No | Given the wide range of CP activities, the required qualifications will differ. Given the wide range of pre-graduate education in pharmacy in Europe, what constitutes basic and advanced training will also differ. In the revised rationale, we therefore specifiy that pharmacists must be “appropriately qualified”, but consider it outside the scope of this work to specify exactly what this means. |
| Should mention Registered Pharmacy Technicians in addition to pharmaceutical staff. | No | The revised definition limits CP providers to pharmacists, who may be supported by other staff. Pharmacy technicians are considered only one among other possible staff who may support pharmacists in conducting CP activities. |
| Clinical pharmacists have to be also experts in communication. | Partly/  unclear | The revised definition includes counselling within the scope of CP activities. In the revised rationale, we therefore specifiy that pharmacists must be “appropriately qualified”, but consider it outside the scope of this work to specify exactly what this means. |
| Also by Internet services ( by intelligent care robots) to targeted patient groups who have the ultimate control of complete data sets, in many cases not available to clinical pharmacists using incomplete or incorrect patient files …..? | Yes | The revised definition limits CP providers to pharmacists. The revised rationale clarifies that CP activities may be supported by other staff or tools, but such supportive activities would not be considered CP in their own right. |
| I think it is important to look at what has been written by Steve Hudson: “Pharmaceutical care is a social need representing the level of quality of care with medicines that is perceived by the patient(s) and not simply a definition of the profession of pharmacy. | Yes | We agree that PC may be both, an expression of a patient need and a professional response to address that need, depending on whether PC is interprteted as “care around pharmaceuticals” or as “care by pharmacists”. The destinction is of obvious relevance to the decision as to who can provide PC.  Nevertheless, our aim here is to define *Clincal Pharmacy*, and in clarifying its relationship to PC, we took the pragmatic choice to use the official PCNE definition (which is the result of a comprehensive consensus process) as our point of reference, which interprets PC in the latter sense, i.e. as “the contribution of pharmacists …”. In comparison to PC, the term CP more clearly refers to a professional practice/discipline than to a patient need (i.e. even the statement that ‘a patient has a need for clinical pharmacy’ would refer to a professional activity) and this is reflected by the revised CP definition and rationale presented here. Nevertheless, CP’s specified aim of “achieving patient-centered goals” should clarify that at individual patient level, CP activities are conducted *in response* to patient needs. |
| The term pharmaceutical staff must be more specific to explain which part of the staff in the pharmacy can deliver the service. Can technical staff provide these services? | Yes | The revised definition limits CP providers to pharmacists. The revised rationale clarifies that CP activities may be supported by other staff or tools, but such supportive activities would not be considered CP in their own right. |
| It is not developed if clinical pharmacists should have a specific clinical education or training. | No | In the revised rationale, we specifiy that pharmacists must be “appropriately qualified”, but consider it outside the scope of this work to specify exactly what this means. |
| I expected clinical pharmacist profession mentioned in the definition. | No | To our knowledge “clinical pharmacist” is not a protected professional title, which is restricted to pharmacists with particular training. |
| It is not clear if providing clinical pharmacy service requires a certificate or in other words a specialisation in a university. | Partly/  unclear | In the revised rationale, we specifiy that pharmacists must be “appropriately qualified”, but consider it outside the scope of this work to specify exactly what this means. |
| What about a pharmacy technician doing medication reconciliation, or nurse or physician? | Yes | The revised definition limits CP providers to pharmacists. The revised rationale clarifies that CP activities may be supported by other staff or tools, but such supportive activities would not be considered CP in their own right. |
| In my mind, clinical pharmacy should be provided by pharmacists and Only in the very last sentence the word pharmacist is mentioned, but I think it is OK. | Yes | The revised definition limits CP providers to pharmacists. The revised rationale clarifies that CP activities may be supported by other staff or tools, but such supportive activities would not be considered CP in their own right. |
| Pharmacy technician can provide clinical pharmacy services? To me it is not clear. | Yes | The revised definition limits CP providers to pharmacists. The revised rationale clarifies that CP activities may be supported by other staff or tools, but such supportive activities would not be considered CP in their own right. |
| "Pharmacy professionals"? or Pharmacists, which I'd support. | Yes | The revised definition limits CP providers to pharmacists. The revised rationale clarifies that CP activities may be supported by other staff or tools, but such supportive activities would not be considered CP in their own right. |
| I think that medical doctors and trained nurses can - and do - provide also clinical services serving the same aims. | No | The revised rationale explicitly clarifies this point. It is acknowledged that non-pharmacy health care professionals can and do engage in activities that aim to “optimise the utilisation of medicines” …, but the definition reflects our view that such practices are only consistent with the term Clinical *Pharmacy* if they are conducted by pharmacists. E.g. medical doctors would be unlikely to agree that by reviewing and adjusting medication they are practicing “clinical pharmacy”. |
| Maybe a note should be made to distinguish between pharmacy staff and pharmaceutical staff. E.g. an admin person is part of the pharmacy staff but this person is not pharmaceutical staff although he/she is working under the responsibility of the pharmacist. | Yes | The revised definition and rationale now avoid the potentially ambiguous terms pharmacy staff and pharmaceutical staff. The revised definition limits CP providers to pharmacists. The revised rationale clarifies that CP activities may be supported by other staff or tools, but such supportive activities would not be considered CP in their own right. |
| See before, i think everybody can do it not only pharmaceutical staff, see above. | No | The revised rationale explicitly clarifies this point. It is acknowledged that non-pharmacy health care professionals can and do engage in activities that aim to “optimise the utilisation of medicines” …, but the definition reflects our view that such practices are only consistent with the term Clinical *Pharmacy* if they are conducted by pharmacists. E.g. medical doctors would be unlikely to agree that by reviewing and adjusting medication they are practicing “clinical pharmacy”. |
| It is important to keep clinical pharmacy under the responsibility of pharmacists. | Yes | The revised definition limits CP providers to pharmacists. The revised rationale clarifies that CP activities may be supported by other staff or tools, but such supportive activities would not be considered CP in their own right. |
| Yes, but i dont think this is very important. | Yes |  |
| Registered Pharmacy Technicians. | No | The revised definition limits CP providers to pharmacists. The revised rationale clarifies that CP activities may be supported by other staff or tools, but such supportive activities would not be considered CP in their own right. |
| Also by Internet services ( by intelligent care robots) to targeted patient groups who have the ultimate control of complete data sets, in many cases not available to clinical pharmacists using incomplete or incorrect patient files …..? By the revolution of intelligent internet services provided by those companies who have the trust of patients, whereas the patients are in control who to give the access to their complete clinical data set. The circumstances and technical possibilities are changing more rapidly than we can envisage. ESCP has to think about that. | Yes | The revised definition limits CP providers to pharmacists. The revised rationale clarifies that CP activities may be supported by other staff or tools, but such supportive activities would not be considered CP in their own right. |
| I think that these services should be offered only by pharmacists. | Yes | The revised definition limits CP providers to pharmacists. The revised rationale clarifies that CP activities may be supported by other staff or tools, but such supportive activities would not be considered CP in their own right. |
| In my opinion, (specialised) nurses or doctor's should be as able to provide clinical pharmacy services as pharmacy staff. Nevertheless, for pharmacy staff this will be core business as opposed to medical staff. | No | The revised rationale explicitly clarifies this point. It is acknowledged that non-pharmacy health care professionals can and do engage in activities that aim to “optimise the utilisation of medicines” …, but the definition reflects our view that such practices are only consistent with the term Clinical *Pharmacy* if they are conducted by pharmacists. E.g. medical doctors would be unlikely to agree that by reviewing and adjusting medication they are practicing “clinical pharmacy”. |
| Like in question 3. It's not specified if a specific education or training is mandatory for the exercice of clinical pharmacy. In Switzerland, like in many other european countries, pregraduate education (Pharmacy at the University) is not sufficient to acquire all skills for clinical pharmacy. In Switzerland, we have a postgraduate practicale education of 18 months for clinical pharmacy, achieved in a ward. | Partly/  unclear | In the revised rationale, we specifiy that pharmacists must be “appropriately qualified”, but consider it outside the scope of this work to specify exactly what this means. |
| It seems that "registered pharmacist" is too wide definition for that. Not all registered pharmacist can provide the service, or do you mean that licence to provide (or not to provide) clinical pharmacy service is settled by pharmacist's work contract provisions? | Partly/  unclear | In the revised rationale, we specifiy that pharmacists must be “appropriately qualified”, but consider it outside the scope of this work to specify exactly what this means. |
| No, a disease nurse or a phasician can provide clinical pharmacy services as well, yet we still should call them clinical pharmacy (services). | No | The revised rationale explicitly clarifies this point. It is acknowledged that non-pharmacy health care professionals can and do engage in activities that aim to “optimise the utilisation of medicines” …, but the definition reflects our view that such practices are only consistent with the term Clinical *Pharmacy* if they are conducted by pharmacists. E.g. medical doctors would be unlikely to agree that by reviewing and adjusting medication they are practicing “clinical pharmacy”. |
| Only pharmacists. | Yes | The revised definition limits CP providers to pharmacists. The revised rationale clarifies that CP activities may be supported by other staff or tools, but such supportive activities would not be considered CP in their own right. |
| Should be every trained pharmacist (not technician) who is working in both community pharmacy en hospital pharmacy. | Yes | In the revised rationale, we specifiy that pharmacists must be “appropriately qualified”, but consider it outside the scope of this work to specify exactly what this means. |
| 1. **In which settings can Clinical Pharmacy services be provided?** |  |  |
| For those not familiar with the term "clinical" will probably always refer to a "clinic". | Yes | The revised definition explicitly states that CP practice may be conducted regardless of the setting. The rationale clarifies that “the word “Clinical” therefore refers to the focus or orientation of CP activities (i.e. patients rather than pharmaceutical products) and not the setting in which they are provided”. |
| (Maybe adding some examples of settings...). | No | Given that the revised definition states regardless of setting, we do not consider giving examples as necessary. On the contrary, providing examples may raise questions as to whether example settings that are not named are included. |
| In every setting where pharmaceutical care is delivered. | Yes | The revised definition explicitly states that CP practice may be conducted regardless of the setting. The rationale clarifies that “the word “Clinical” therefore refers to the focus or orientation of CP activities (i.e. patients rather than pharmaceutical products) and not the setting in which they are provided”. |
| And I do think that clinical pharmacy can be provided in other settings than institutions, so it is important that you do not restrict it. | Yes | The revised definition explicitly states that CP practice may be conducted regardless of the setting. The rationale clarifies that “the word “Clinical” therefore refers to the focus or orientation of CP activities (i.e. patients rather than pharmaceutical products) and not the setting in which they are provided”. |
| Any setting probably is not clear enogh. The current definition on our website uses two sentences to explain this issue. | Yes | The revised definition explicitly states that CP practice may be conducted regardless of the setting. The rationale clarifies that “the word “Clinical” therefore refers to the focus or orientation of CP activities (i.e. patients rather than pharmaceutical products) and not the setting in which they are provided”. |
| I do not agree with specifying settings where CP can be delivered. | Yes | The revised definition explicitly states that CP practice may be conducted regardless of the setting. The rationale clarifies that “the word “Clinical” therefore refers to the focus or orientation of CP activities (i.e. patients rather than pharmaceutical products) and not the setting in which they are provided”. |
| But again, do you need to add the setting. Does a Surgeon have to explain he works in an operating theatre? | Yes | It is an issue of ongoing controversy as to whether the word “Clinical” refers to the focus or orientation of CP activities (i.e. patients rather than pharmaceutical products) or the setting in which they are provided. We therefore think it is important to clarify this here. |
| The seeting needs to be broad- in all enviroments in with medicines are prescribed and use for public/ patient benefit. | Yes | The revised definition explicitly states that CP practice may be conducted regardless of the setting. |
| Again depending on the definitions the content and who is performing is much more important than the setting. | Yes | It is an issue of ongoing controversy as to whether the word “Clinical” refers to the focus or orientation of CP activities (i.e. patients rather than pharmaceutical products) or the setting in which they are provided. We therefore think it is important to clarify this here. |
| Models proposing that pharmacists could be employed by medical doctors to provide such services should be excluded by definition. Not fully, but enough. | No | The revised definition explicitly states that CP practice may be conducted regardless of the setting. We do not agree with the exclusion of such care models because we do not consider them unethical per se. |
| Now it's to generic, should be specified more No, therefore not addressed. | No | The revised definition explicitly states that CP practice may be conducted regardless of the setting. |
| For emphasis, I would have preferred a wider inclusion of ambulatory-based activities, e.g. nursing homes, home care organizations, etc. | No | The revised definition explicitly states that CP practice may be conducted regardless of the setting. We therefore do not consider it necessary to provide examples. |
| I presume it can be anywhere? | Yes | The revised definition explicitly states that CP practice may be conducted regardless of the setting. |
| It does but that does not result from the survey results that clearly indicated it is restricted to hospital. Extension says: "Clinical Pharmacy services can be delivered in any setting". I would have written: "Clinical pharmacy services can be provided in any context where the use of a drug is expected". | Partly/  unclear | This is a misunderstanding. In the survey, approximately half believed that CP services could be provided in any private or public space, ~70% stated that CP could be provided in community pharmacies. |
| 'Any setting' is not specific. On purpose, therefore not addressed. | Yes | The revised definition explicitly states that CP practice may be conducted regardless of the setting. |
| From my point of view, the expression "in any setting" is too generic. Definition needs to mention that the clinical pharmacy services can be provided irrespective the setting since there is a lot of confusion about this and as society we want to embrace hospital and community pharmacists as well as pharmacists working in other environments such as homes for the elderly for example. | Yes | The revised definition explicitly states that CP practice may be conducted regardless of the setting. As the revised rationale states, “the word “Clinical” refers to the focus or orientation of CP activities (i.e. patients rather than pharmaceutical products) and not the setting in which they are provided”. |
| In the rational you said that the setting does not matter. | Yes | The revised definition explicitly states that CP practice may be conducted regardless of the setting. |
| These services can be provided outside the pharmacy? In the ward or department? In the community pharmacies? Probably we need to define the settings. | No | The revised definition explicitly states that CP practice may be conducted regardless of the setting. We therefore do not consider it necessary to provide examples. |
| Every setting. | Yes | The revised definition explicitly states that CP practice may be conducted regardless of the setting. We therefore do not consider it necessary to provide examples. |
| Clinical pharmacy does not have to be in ONLY direct patient care settings e.g. working in a Medicines information centre (as a NON-direct patient care setting) can be a Clinical pharmacy setting in my view. | Yes | The revised definition explicitly states that CP practice may be conducted regardless of the setting. We therefore do not consider it necessary to provide examples. |
| There are some facilities needed such as information on drug intake, laboratory measures, indications, contra indications etc. | No | We agree but given our aim of defining CP we consider this beyond the scope of this work. |
| Last sentence should be more explicit, reading: "The word “Clinical” therefore refers to the focus of pharmaceutical activities and not to the setting in which they are provided. | Yes | The revised rationale states that “The word “Clinical” therefore refers to the focus or orientation of CP activities (i.e. patients rather than pharmaceutical products) and not the setting in which they are provided“. |
| Unclear rationale. | Yes | The revised rationale states that “The word “Clinical” therefore refers to the focus or orientation of CP activities (i.e. patients rather than pharmaceutical products) and not the setting in which they are provided“. |
| Needs clarity. | Yes | The revised rationale states that “The word “Clinical” therefore refers to the focus or orientation of CP activities (i.e. patients rather than pharmaceutical products) and not the setting in which they are provided“. |
| In french, "clinique" means either "directly, close to the patients" or "care institution". I thing that in english, there is only the second meaning. According to the first meaning, clinical pharmacy can be done in every setting, but not according the second meaning. I'm a bit reluctant to use "clinical pharmacy" for community pharmacy, but I'm ok for small first line facilities (dispensary, ...). If the definition is not changed, i think that the rationale should refer to Latin ethymology. | Yes | The revised rationale states that “The word “Clinical” therefore refers to the focus or orientation of CP activities (i.e. patients rather than pharmaceutical products) and not the setting in which they are provided“. |
| The definition is very clear, but I dont think this is very important. | Yes | It is an issue of ongoing controversy as to whether the word “Clinical” refers to the focus or orientation of CP activities (i.e. patients rather than pharmaceutical products) or the setting in which they are provided. We therefore think it is important to clarify this here. |
| If by 'view' you mean the respondents' view then No. Clinical pharmacy services can be provided in any setting including GP practice and community setting. | Yes | The revised rationale states that “The word “Clinical” therefore refers to the focus or orientation of CP activities (i.e. patients rather than pharmaceutical products) and not the setting in which they are provided“. |
| I think that community pharmacies can be also a setting where Clinical Pharmacy Services can be provided. | Yes | The revised rationale states that “The word “Clinical” therefore refers to the focus or orientation of CP activities (i.e. patients rather than pharmaceutical products) and not the setting in which they are provided“. |
| In adition to community and hospital pharmacies, I consider nursing homes and patients' homes, as well as public health programme settings appropriate, but not physician's practices. | Yes | The revised rationale states that “The word “Clinical” therefore refers to the focus or orientation of CP activities (i.e. patients rather than pharmaceutical products) and not the setting in which they are provided“. |
| The current definition leaves room for providing services outside a setting, eg online etc. | Yes | The revised rationale states that “The word “Clinical” therefore refers to the focus or orientation of CP activities (i.e. patients rather than pharmaceutical products) and not the setting in which they are provided“. |
| Again, more emphasis on ambulatory care is in my opinion desirable, specifically because of the demographic development and an increasing shift from inpatient ot ambulatory care. | Yes | The revised rationale states that “The word “Clinical” therefore refers to the focus or orientation of CP activities (i.e. patients rather than pharmaceutical products) and not the setting in which they are provided“. |
| I think that Clinical pharmacy services can be provided in any context where the use of a drug is expected. | Yes | The revised rationale states that “The word “Clinical” therefore refers to the focus or orientation of CP activities (i.e. patients rather than pharmaceutical products) and not the setting in which they are provided“. |
| For sure they can be conducted anywhere, including (and especially in) a physicians practice. | Yes | The revised rationale states that “The word “Clinical” therefore refers to the focus or orientation of CP activities (i.e. patients rather than pharmaceutical products) and not the setting in which they are provided“. |
| Still not very specific, would 'in a health care setting' not be sufficient and required? | Yes | The revised rationale states that “The word “Clinical” therefore refers to the focus or orientation of CP activities (i.e. patients rather than pharmaceutical products) and not the setting in which they are provided“. |
| Clinical Pharmacy services can also be provided in community pharmacies and at nursing homes. | Yes | The revised rationale states that “The word “Clinical” therefore refers to the focus or orientation of CP activities (i.e. patients rather than pharmaceutical products) and not the setting in which they are provided“. |
| 1. **What is the relationship between Clinical Pharmacy and Pharmaceutical Care?** |  |  |
| Also, pharmaceutical care is both (a professional practice and a scientific discipline). | Yes | The revised definition avoids the potentially ambiguous term “scientific” and distinguishes between activities that constitute practice on the one hand and research to inform that practice. While it is possible to conduct research to evaluate PC, we consider the term “care” as reflecting practice rather than knowledge generation (i.e. research). This seems consistent with the PCNE definition of PC, which specifies that PC targets individual patients. In contrast, research usually targets *groups* of patients. As reflected in the diagram, we therefore consider PC to be informed by research, but not constituting a research discipline in its own right. |
| Clinical Medication Review, which is mostly being used in European context, is based on the two aspects of PC practice. I am not sure that the PCNE definition of PC delivery is paying enough attention to the quality of care as perceived by the patient(s). As a result, I am not sure whether the Clinical Pharmacy practice and PC delivery are synonymous. | Yes | The revised definition avoids the term synonymous, because such a statement would imply assumptions of the term PC beyond its published definition by PCNE. As far as CP is concerned, the quality of care as perceived by the patient is addressed by the term “patient centerd goals” and in the rationale by the statement that “health-related goals in a way that respects human dignity and personal choice”. |
| Need to include the term patients in this definition (the word patient is only mentioned 3 times. | Yes | The proposed definition characterizes CP practice as being patient rateher than product centered and as aiming to achive patient centered goals. |
| Pharmaceutical care is a subset of clinical pharmacy, this is not clear in the definition. | Yes | The revised definition clarifies that CP practice encompasses but is not limited to PC care models. The figure should make it clear that PC is a subset of CP. |
| Every professional practice is based on a specific and complex knowledge domain. Now it seems that Pharmaceutical care delivery might not be based on an academic discipline. | Yes | The definition extension and figure make clear that CP practice is informed by knowledge generated through research. PC is defined as a subset of CP practice and as such is informed by the same knowledge base. This also explicitly mentioned in the rationale. |
| Not very clear how they differ apart from the fact that pharmaceutical care targets individuals and clinical pharmacy can be on a population level. To me they are quite synonymous as stated at the end of the extension. | Yes | The comment is in agreement with the view expressed in the revised definition. |
| Little confusing here as Clinical Pharmacy practice and Pharmaceutical Care delivery are considered synonymous. | Yes | We clarify in the rationale that: we see CP as a broader concept than PC because CP practice additionally comprises activities that are predominantly targeted at providers, such as the development and dissemination of clinical practice guidelines (eg in the context of antibiotic stewardship). |
| It seems thus that pharmaceutical care does not have any scientific status - and that is not true! | Yes | The definition extension and figure make clear that CP practice is informed by knowledge generated through research. PC is defined as a subset of CP practice and as such is informed by the same knowledge base. This is also explicitly mentioned in the rationale. |
| According to the extension, the only difference is that clinical pharmacy can be delivered on populion level, while pharmaceutical care is only at an individual level. However, pharmaceutical care includes activities of the pharmacist not directly related to specific medications (but medicines use in general), whereas I have the understanding that clinical pharmacy services always target the specific medications used (or not used) by an individual. No, not according to our definition. | Yes | The figure should make it clear that PC is a subset of CP. CP therefore encompasses all aspects of PC while not being limited to care models that classify as PC. |
| It's a play around of CP services and CP practice vs PC whilst this question is asking about CP. Is services or practice meant in this question? | Yes | The revised definition avoids the term service. The relationship between CP research, CP practice and PC should be clear from the figure and the rationale. PC is a subset of CP practice and is informed by CP research. |
| Does pharmceutical care englobes clincal pharmacy? | Yes | No, the other way around. The revised definition and rationale sould make it clear that PC is seen as a subset of CP practice and is informed by CP research. |
| Clinical pharmacy is not only in individual patients. | Yes | We clarify in the rationale that: we see CP as a broader concept than PC because CP practice additionally comprises activities that are predominantly targeted at providers, such as the development and dissemination of clinical practice guidelines (eg in the context of antibiotic stewardship). |
| Pharm Care is a wider notion than clinical pharmacy care? | Yes | The revised definition and rationale should make it clear that PC is seen as a subset of CP practice. |
| The fact that Clinical pharmacy practice and Pharmaceutical Care delivery are synonymous has been made explicit. The difference between Clinical Pharmacy and Pharmaceutical Care can be quite easily derived from the extension, but is not made explicit. | Yes | The rationale should make this point clear: “However, we see CP as a broader concept than PC because CP practice additionally comprises activities that are predominantly targeted at providers, such as the development and dissemination of clinical practice guidelines (eg in the context of antibiotic stewardship)”. |
| It does not become clear wether Clinical pharmacy is broader than pharmaceutical care. | Yes | The revised rationale should make this point clear: “However, we see CP as a broader concept than PC because CP practice additionally comprises activities that are predominantly targeted at providers, such as ….”. |
| In the proposed extension of the definition, it is suggested that clinical pharmacy and pharmaceutical care ar the same, namely targetting individual patients. However, the definition of pharmaceutical care (originally by Strand, also by PCNE) might be broader, also including activities at the drug process level. Clinical pharmacists cannot provide recommendations or counsel patients without guidelines e.g. and the drug process (prescribving, delevering, preparing, administration, monitoring,...) should be organised in a safe way for which a lot of work is done by clinical pharmacists but not on the patient level. So in my opinion, pharmaceutical care is the total of both clinical pharmacy activities at the individual AND at the drug process level. Drug process level: formulary, antibiotic policy, guidelines, analysis of reported medication errors, safe electronic prescribing, organisation of seamless pharmaceutical care etc. is still in the definition. | Yes | The PCNE definition clearly identifies PC as targeting individual patients: “Pharmaceutical Care is the pharmacist’s contribution to the care of *individuals* in order to optimise medicines use and improve health outcomes”. The revised definition reflects our view that activities targeting the medication use process (and therefore populations of patients rather than individuals) fall under the umbrella of CP but (consistent with the PCNE definition) not under PC. |
| The definition was very clear until I read the last sentence. | Yes | The revised definition avoids the apparently confusing notion of CP and PC being synonymous as far as care for individuals concerned but not as far as other aspects of CP research and practice are concerns. |
| I think Clinical Pharmacy comprises also services without a follow-up to determine the impact of the service (like vaccination programs) possible in this definition. | No | The revised rationale clarifies that “The technical acts of compounding or physically administering pharmaceutical products does not fall within the scope of CP practice.” |
| It only mentions where it is synonymous. | Yes | The revised rationale also explicitly states where CP and PC differ: “However, we see CP as a broader concept than PC because CP practice additionally comprises activities that are predominantly targeted at providers, such as ….”. |
| I think this last sentence ending in clinical pharmacy practice and pharmaceutical care delivery are synonymous is quite confusing. I don't really see how clinical pharmacy services and clinical pharmacy practices differ from each other. | Yes | The revised definition avoids the apparently confusing notion of CP and PC being synonymous as far as care for individuals concerned but not as far as other aspects of CP research and practice are concerns. |
| Understandable from the figure, but is "care" fully covering the clinical pharmacy's purpose to make optimal therapeutic drug use? | Yes | According to the PCNE definition, PC encompasses the pharmacist contribution to the care of individuals without restriction. The rationale clarifies that PC as a subset of CP encompasses both cognitive and counselling activities. |
| The last four lines are not understandable at all. The rationale is clearer. I support the appraoch in figure 1. However, please consider that ecnomical advice is included in pharmaceutical care but not in Clinical pharmacy (as a discipline different from pharmacoeconomics), the figure and definitions will have to adress that pharmaceutical care has this additional economic advice aspects to patients. Do not agree. | No | The revised definition avoids the apparently confusing notion of CP and PC being synonymous as far as care for individuals concerned but not as far as other aspects of CP research and practice are concerned. The revised definition clarifies that PC is a subset of CP. Therefore, if economic advice is part of PC it is also part of CP. |
| From my perspective, this discussion should not be part of a definition but discussed in the position paper. both terms are presented als synonyms in the text. | Yes | The revised definition avoids explicit reference to the related concept of PC. The commonalities and differences between CP and PC are adressed in the rationale. |
| Pharmaceutical care definition is not so clear for me. | No | The definition of PC is not part of the CP definition, but provided in the accompanying rationale. |
| From a clinical pharmacy perspective, yes. I'm still a bit in doubt if providers on pharmaceutical care will be satisfied to be limited to a patient-specific role....addrsesed although it is in the PCNE definition. | Partly/  unclear | The patient specific role is consistent with the PCNE definition of Pharmaceutical Care: “Pharmaceutical Care is the pharmacist’s contribution to the care of individuals in order to optimise medicines use and improve health outcomes”. |
| This aspect is contradictory in different sections of the position paper. | Yes | The revised definition avoids the apparently confusing notion of CP and PC being synonymous as far as care for individuals concerned but not as far as other aspects of CP research and practice are concerned. We have checked the definition and rationale for consistency. |
| ...as it states, that both are similar, which I am not too sure of. | Partly/  unclear | The revised definition avoids the apparently confusing notion of CP and PC being synonymous as far as care for individuals concerned but not as far as other aspects of CP research and practice are concerned. The revised rationale clarifies that PC is a subset of CP and that CP practice in contrast to PC additionally comprises activities that are not targeted at individual patients. |
| The mentioned definition is about the concept of a type of care that can be delivered by pharmacists. Not about a 'service'. | Yes | The revised definition avoids the notion of a service but instead refers to CP “practice” and care models. |
| Not sure/clear if the definition is now made to mean that both terms are the same. It mentions where both terms are similar but not where they are different if this would be the case. | Yes | The revised rationale clarifies that PC is a subset of CP and that CP practice in contrast to PC additionally comprises activities that are not targeted at individual patients. |
| The definition of PCNE mentions: "The pharmacist’s contribution...", but in some countries pharmacutical care could be delivered by others. | Partly/  unclear | We took the pragmatic decision to use the PCNE definition because it is based on consensus of PCNE members from a number of European countries. |
| "Are synonymous", is stated. | Yes | The revised rationale clarifies that PC is a subset of CP and that CP practice in contrast to PC additionally comprises activities that are not targeted at individual patients. |
| The services and deliverables model is dependend on the political economic perspective on how healthcare should be organized and will compromise pharmacists’ ambition to the foundation of a sustainable humanistic professional practice. Care and knowledge are not deliverables. | Yes | The revised definition avoids the notion of a service but instead refers to CP “practice” and care models. |
| In my view, the circle for Pharmaceutical Care in figure 1 should not be completely inside of that of Clinical Pharmacy as there might be Pharmaceutical Care activities not necessarily considered Clinical Pharmacy Practice (e.g. discussing non-pharmacological options to treat insomnia in a community pharmacy setting could be considered as pharmaceutical care, but not clinical pharmacy). | Partly/  unclear | It is our view and the definition reflects that CP targets the appropriate use of medicines by patients and professionals, including counselling and self-medication. This includes situations where the use of medicines is not the best available option and other options may be more suitable. We therefore do not see a distinction between PC and CP in this regard. |
| Responsibility is key; I also miss this in PCNE contribution. | Yes | The revised definition states that Clinical Pharmacy practice encompasses (but is not limited to) care models in which pharmacists assume responsibilty for achieving person-centered goals for individual patients as part of a multidisciplinary team. The revised rationale expands on this. |
| If by 'view' you mean the respondents' view, then No. Clinical pharmacy is much broader. | Yes | The revised rationale clarifies that PC is a subset of CP and that CP practice in contrast to PC additionally comprises activities that are not targeted at individual patients. |
| I think that Clinical Pharmacy is an umbrella which encompasses pharmaceutical care. | Yes | The revised rationale clarifies that PC is a subset of CP and that CP practice in contrast to PC additionally comprises activities that are not targeted at individual patients. |
| I think in the rational that is very well and clearly described and I fully agree with this, but I think the chosen wording in the extended definition is rather unclear and in my opinion does not reflect the explanation in the rational. | Yes | The revised definition avoids the apparently confusing notion of CP and PC being synonymous as far as care for individuals concerned but not as far as other aspects of CP research and practice are concerned. The revised rationale clarifies that PC is a subset of CP and that CP practice in contrast to PC additionally comprises activities that are not targeted at individual patients. |
| Clinical pharmacists know what they talk about but a public definition aims at an audience not necessary familiar with theses aspects of pharmacy practice. Therefore, a more "neutral" term like "medication management" should be taken into consideration. | No | While we cannot exclude that medication management is a more intuitively understood term, our aim here was to define Clinical Pharmacy. |
| From a clinical pharmacy perspective: yes! However, pharmaceuticla care seems pretty limited when constrained to patient-specific care (prevention, for example, might also be a population-based task....). | Partly/  unclear | The patient specific role is consistent with the PCNE definition of Pharmaceutical Care: “Pharmaceutical Care is the pharmacist’s contribution to the care of individuals in order to optimise medicines use and improve health outcomes”. |
| Not sure it does differentiate so can't agree/disagree. | Partly/  unclear | The revised rationale clarifies that PC is a subset of CP and that CP practice in contrast to PC additionally comprises activities that are not targeted at individual patients. |
| Yes, pharmaceutical care needs a patient involved. | Yes | The patient specific role is consistent with the PCNE definition of Pharmaceutical Care: “Pharmaceutical Care is the pharmacist’s contribution to the care of individuals in order to optimise medicines use and improve health outcomes”. |
| I would agree that pharmaceutical care is part of clinical pharmacy, but not identical. | Yes | The revised rationale clarifies that PC is a subset of CP and that CP practice in contrast to PC additionally comprises activities that are not targeted at individual patients. |
| I read it as for individual patients they are the same, but that Clinical pharmacy is more than Pharmaceutivcal care. | Yes | The revised definition avoids the apparently confusing notion of CP and PC being synonymous as far as care for individuals concerned but not as far as other aspects of CP research and practice are concerned. The revised rationale clarifies that PC is a subset of CP and that CP practice in contrast to PC additionally comprises activities that are not targeted at individual patients. |
| Are they synonymous or not? | Yes | The revised definition avoids the apparently confusing notion of CP and PC being synonymous as far as care for individuals concerned but not as far as other aspects of CP research and practice are concerned. The revised rationale clarifies that PC is a subset of CP and that CP practice in contrast to PC additionally comprises activities that are not targeted at individual patients. |
